# High-resolution chronologies of anthropogenic soil substrates based on portable luminescence reader data

**DOI:** 10.1038/s41598-025-29066-3

**Published:** 2025-11-27

**Authors:** Dominik Brill, W. Marijn van der Meij, Paula von Lengrießer, Frederike Tschernich, Anja Zander, Stephan Opitz, Tony Reimann

**Affiliations:** Institute of Geography, University of Cologne, 50674 Cologne, Germany

**Keywords:** Soil erosion, Plaggic anthrosol, Luminescence dating, Holocene, Anthropocene, Germany, Colluvium, Ecology, Ecology, Environmental sciences

## Abstract

Anthropogenic soil erosion and plaggen agriculture have shaped European agricultural landscapes for millennia. The anthropogenic soil substrates from these practices, such as colluvium and plaggen layers, are valuable geoarchives for reconstructing phases and rates of human activity. However, reconstructions on the landscape scale are limited by the small number of high-resolution chronologies of these substrates. We demonstrate how high-resolution chronologies can be developed for a plaggen soil and a colluvium in western Germany, using a portable optically stimulated luminescence (pOSL) reader, combined with conventional OSL dating and Bayesian age depth modeling. While the centimetre-scale pOSL chronologies provide no significant new insights for the uniformly developed plaggen soil, they reveal dynamic deposition processes in the colluvium that conventional OSL dating missed. Deposition rates in the colluvium varied by orders of magnitude over the last 3500 years, from near-zero deposition during the Migration period to brief episodes or even single events with accelerated deposition during Roman times and Late Middle Ages. These easily obtainable pOSL measurements promise scaling from local to regional contexts, offering new opportunities to reconstruct not just high-resolution chronologies but also spatial patterns of human-induced landscape change – currently beyond the reach of conventional methods.

**Supplementary Information:**

The online version contains supplementary material available at 10.1038/s41598-025-29066-3.

## Introduction

**Fig. 1:**
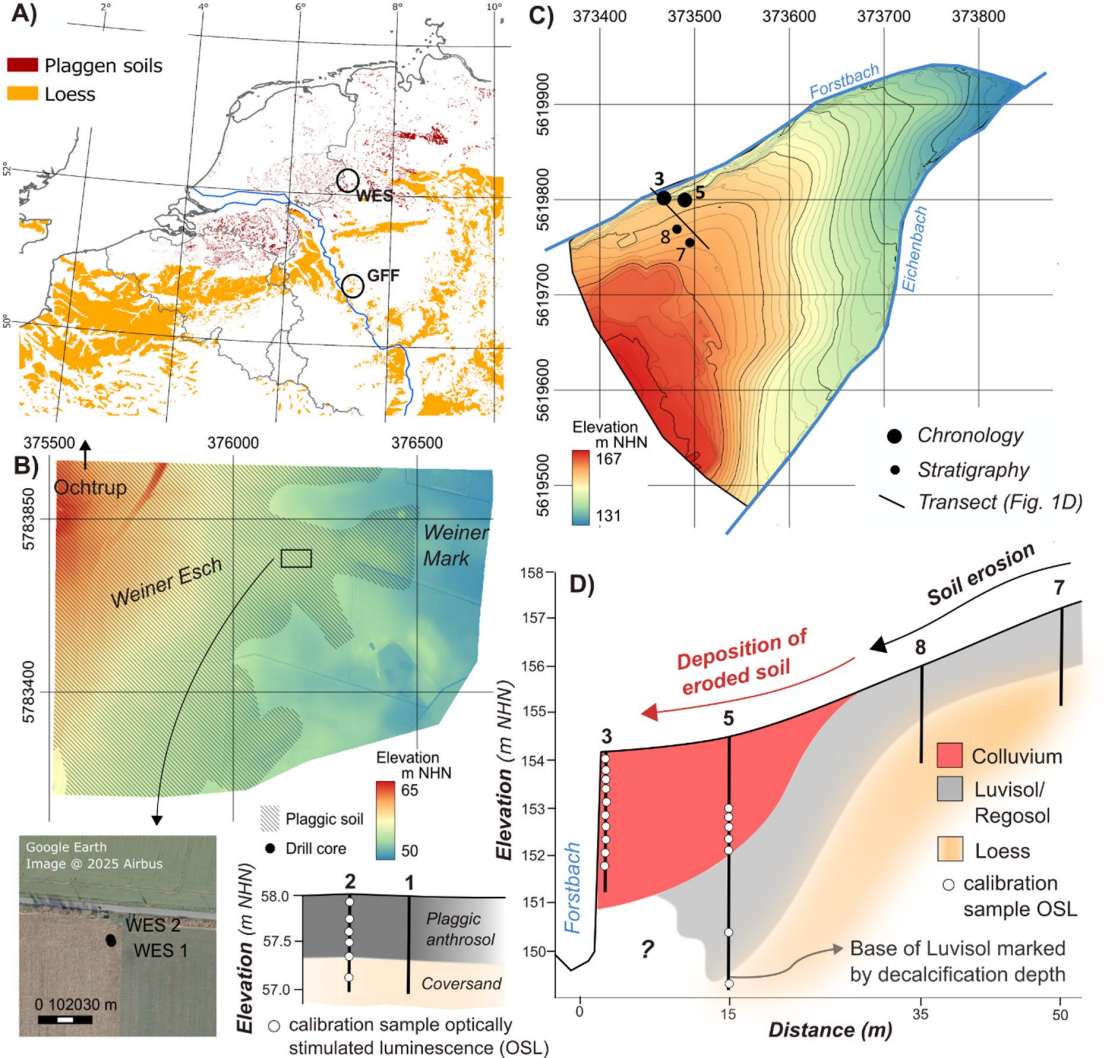
Study area. (**A**) Distribution of plaggen soils (compiled from^26,27,28^) and loess (adopted from^29^) in central Europe with locations of the plaggen site (WES) and the colluvium site (GFF). (**B**) Distribution of plaggen soils^26^, location of sediment cores and subsurface stratigraphy at Weiner Esch. (**C**) Topography of Gut Frankenforst with locations of sediment cores. (**D**) Schematic along-slope stratigraphy at Gut Frankenforst (see (**C**) for transect location) with position of samples for conventional luminescence dating. DEMs in (**B**) and (**C**) based on Geobasis NRWs. Satellite image in (**B**) was taken from Google Earth. Maps in (**A**), (**B**) and (**C**) have been created with QGIS version 3.36.1 (https://download.qgis.org/downloads/).

Humans have significantly impacted Central Europe’s agricultural landscapes by altering natural sediment dynamics, leading to modifications in relief and soils^1^. The first significant human-induced soil erosion occurred around 7500 years ago, particularly in vulnerable loess areas (Fig. 1A)^2^. During the Middle Ages, population growth drove the expansion and intensification of agricultural areas, causing more drastic landscape changes, including relief leveling, soil truncation, and the development of thick colluvial bodies^3,4^. These same drivers also triggered the rapid expansion of plaggen agriculture (Fig. 1A)^5^. Plaggen soils are found in areas with sandy substrates of peri-glacial origin. These poor soils were fertilized using a mixture of humic topsoil sods from areas with more fertile soils and animal dung, forming thick anthropogenic plaggen horizons (also called Esch, es, or enk) over time. The source areas of the topsoil sods were exposed to wind erosion^6^.

Understanding the processes and drivers behind anthropogenic landscape change is crucial for improving numerical models that predict these changes, which in turn inform sustainable soil and land management strategies^7,8^. In this context, high-resolution chronologies of anthropogenic soil substrates are essential for detecting changes in depositional histories, correlating findings across sites, and scaling results to the landscape level^9^. Despite some challenges related to dating anthropogenic soil substrates, numerical dating techniques can provide accurate chronologies by determining the age of incorporated organic material (i.e., radiocarbon dating^10,11^) or the timing of the last sediment remobilization (i.e. luminescence dating^12,13^). Due to the ubiquity of clastic sediment grains, in particular luminescence dating has proven to generate time-resolved records with very good resolution^12,14,15^. However, since these analyses are subject to cost- and time constraints, high-resolution chronologies are rather rare, and both the number of samples per site and the number of dated locations is restricted significantly.

A time- and cost-efficient alternative to traditional dating techniques is provided by portable optically stimulated luminescence (pOSL) reader analysis^16,17^. Conventional OSL dating requires extensive laboratory resources to quantify the burial dose (the accumulated energy in purified quartz or feldspar extracts) and the dose rate (the accumulation rate of energy), resulting in considerable costs and analysis times of days to weeks per sample^18^. By determining luminescence signals from unprocessed bulk soil samples, pOSL readers can reduce analysis time per sample to a couple of minutes^17^. Although the simplified measurement procedure reduces dating accuracy and precision, as other factors besides age – such as dosimetry, sediment composition and luminescence signal resetting – may influence the luminescence signal^16^, pOSL has proven its potential as a proxy for deposition age. When calibrated against independent datings, pOSL has produced robust chronologies in settings where these other factors are uniform across samples, such as dune fields^19,20^, beach-ridge plains^21^, wetlands^22^, and colluvial deposits^23,24^.

In this study, we show that pOSL can be used to develop chronologies for anthropogenic soil substrates with unprecedented resolution, offering unique insights into their evolution. We use pOSL to reconstruct the phases and rates of plaggic Anthrosol formation at the Weiner Esch and colluvium generation at Gut Frankenforst, both in western Germany. We present a standardized workflow for transforming pOSL signals into high-resolution chronologies using conventional OSL dating and Bayesian age modeling, and evaluate the benefits for understanding soil and landscape dynamics.

### Study sites

The first study site, the Weiner Esch (WES) south of Ochtrup, Germany (Fig. 1A), consists of Late Weichselian (MIS 2) cover sands, underlain by Cretaceous clay- and limestones and Saalian (MIS 6 and 8) till and ground moraines^25^. The cover sands are porous and nutrient-poor, leading to the formation of gleyic Podzols as natural soils^26^. Due to plaggen agriculture, more than 10% of the area is currently covered by plaggic Anthrosols, which were sampled using two 1-meter sediment cores from an agricultural field (WES 1 and 2, Fig. 1B).

The second site, Gut Frankenforst (GFF), located at the southern edge of the Lower Rhine Area (Fig. 1A), contains Weichselian loess overlying Miocene volcanic rocks^31^. The natural soils are Luvisols formed in the loess^26^ (Fig. 1D), which have been used for agriculture for thousands of years. As a consequence, the Luvisols are truncated at the upper and middle slopes (cores GFF 7 and 8), with colluvium covering the lower slopes (cores GFF 3 and 5)^32^. We sampled the colluvium from a 5-meter long sediment core (GFF 5) and a nearby 3-meter high outcrop (GFF 3) at the Forstbach (Fig. 1C).

### Workflow

To generate high-resolution chronologies for colluvium and plaggen soils, we performed pOSL measurements on dried, but otherwise unprocessed sediment with centimeter-scale resolution. For each sample, pOSL signals were combined with uncertainties estimated from the instrumental error, sample-internal pOSL scatter, and inter-sample scatter of dose rates and luminescence sensitivity of a set of calibration samples that underwent full luminescence dating. The high-resolution pOSL records were then transformed into ages using transfer functions^19^, constructed by correlating pOSL signals with conventional quartz OSL ages sampled from the same material. Finally, Bayesian modeling, with the simple assumption of increasing ages with depth (Sequence model in OxCal^33^), was applied to generate age-depth models with constrained uncertainties and without age inversions, allowing for the calculation of high-resolution deposition rates (*see methods section for* more *details*).

## Results and interpretation

### Stratigraphy and suitability of substrates for dating with pOSL

**Fig. 2:**
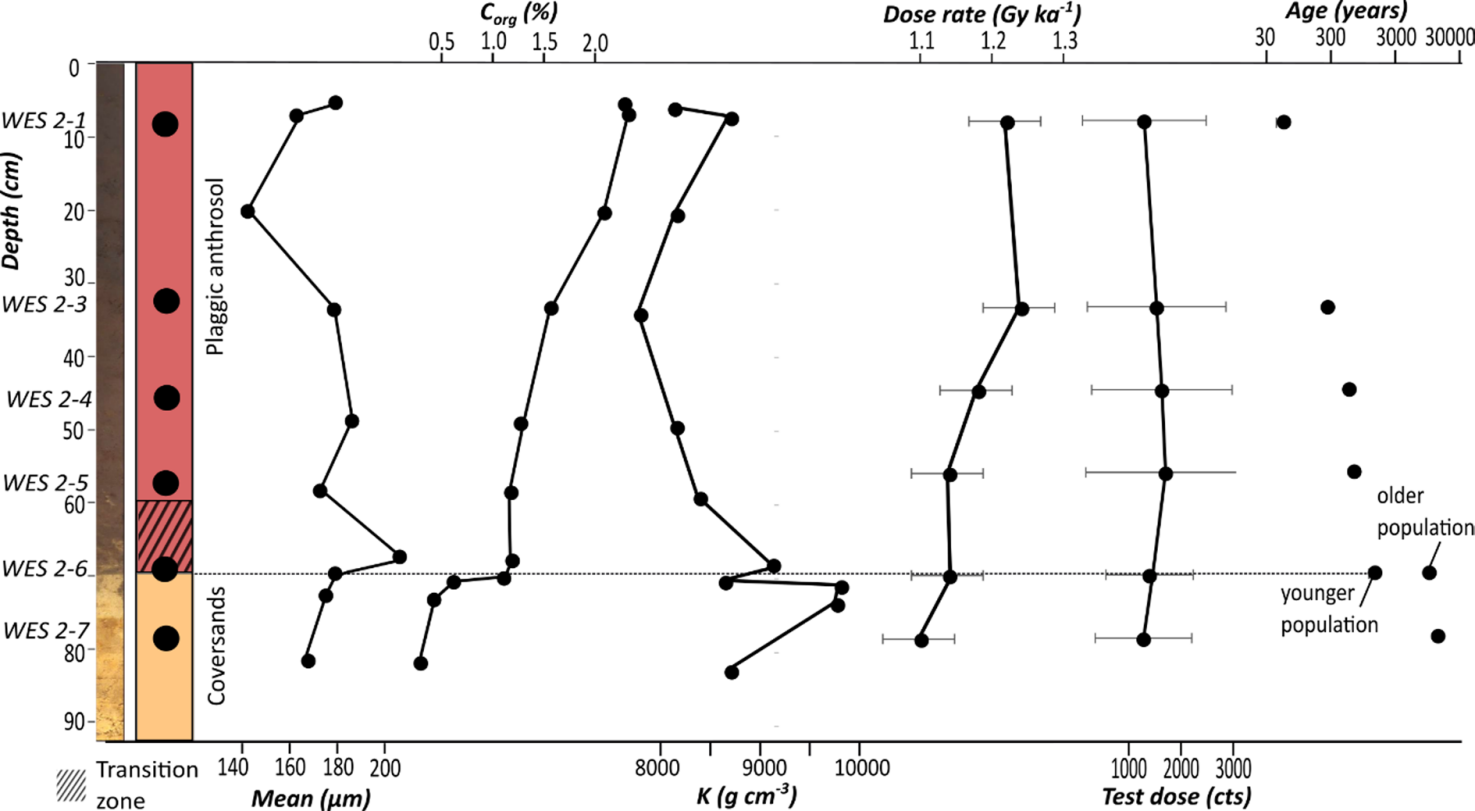
Stratigraphy, sediment composition and luminescence properties for WES 2. Test dose response is a measure of the sample’s luminescence sensitivity. Age uncertainties are masked due to the size of the dots.

Both WES cores reveal a plaggic Anthrosol in their upper 70 cm, overlying cover sands (Fig. 2). The uppermost 25 cm of the plaggen soil show signs of active plowing, while the basal part, between 60 and 70 cm below surface (b.s.), shows intercalated sods and lenses of cover sand not fully homogenized by tillage. Sediment analyses conducted with a resolution of ~ 10 cm reveal overall very homogeneous, sandy grain sizes with mean values between 142 and 186 μm (9% relative standard deviation, RSD) for both plaggen soil and cover sands. Chemical composition is significantly different between the nutrient-poor cover sands and the plaggic Anthrosol with regard to organic carbon and leachable elements, such as phosphorus or magnesium (increased values in the plaggen soil). However, it is homogeneous with respect to elements more important for dose rates such as potassium (7.7% RSD) (Fig. 2, Tab. S2).

The luminescence properties of the sediments were investigated based on six samples from WES 2 (WES 2−1 to 2–6) dated with conventional luminescence of coarse-grain quartz, five from the plaggic Anthrosol and one from the underlying cover sands (Fig. 2). All six samples reveal homogeneous geochemical and luminescence properties that could influence the pOSL signal beside the deposition age, which supports the assumption that deposition age is the main control on the pOSL signal. Dose rates are in a range of 1.10 to 1.24 Gy ka^− 1^ (5.9% RSD) (Fig. 2, Tab. S4), and quartz signal sensitivities (i.e., their signal response to a fixed laboratory dose of ~ 0.5 Gy) show comparably small variability between the samples as well (12% RSD) (Fig. 2). Complete luminescence signal resetting is indicated by moderate scatter (12–30% over-dispersion, OD) and normal distribution of equivalent dose values for five out of six samples (Tab. S5, Fig. S9). These samples date the cover sands to 12,500 ± 600 years, and the plaggic Anthrosol from 50 ± 10 years to 620 ± 50 years (Fig. 2). The only exception is WES 2–6 from the transition zone between cover sands and plaggen soil. Likely due to the influence of mixing between both strata, this sample has a bimodal equivalent dose distribution with a younger grain population reflecting the age of the basal plaggen soil (1,300 ± 200 years), and an older grain population reflecting the upper part of the cover sands (9,100 ± 700 years) (see S4.5 in supplement for details on age calculations). The effect of incomplete signal resetting for WES 2–6 on dating with pOSL is investigated in the following section.

**Fig. 3:**
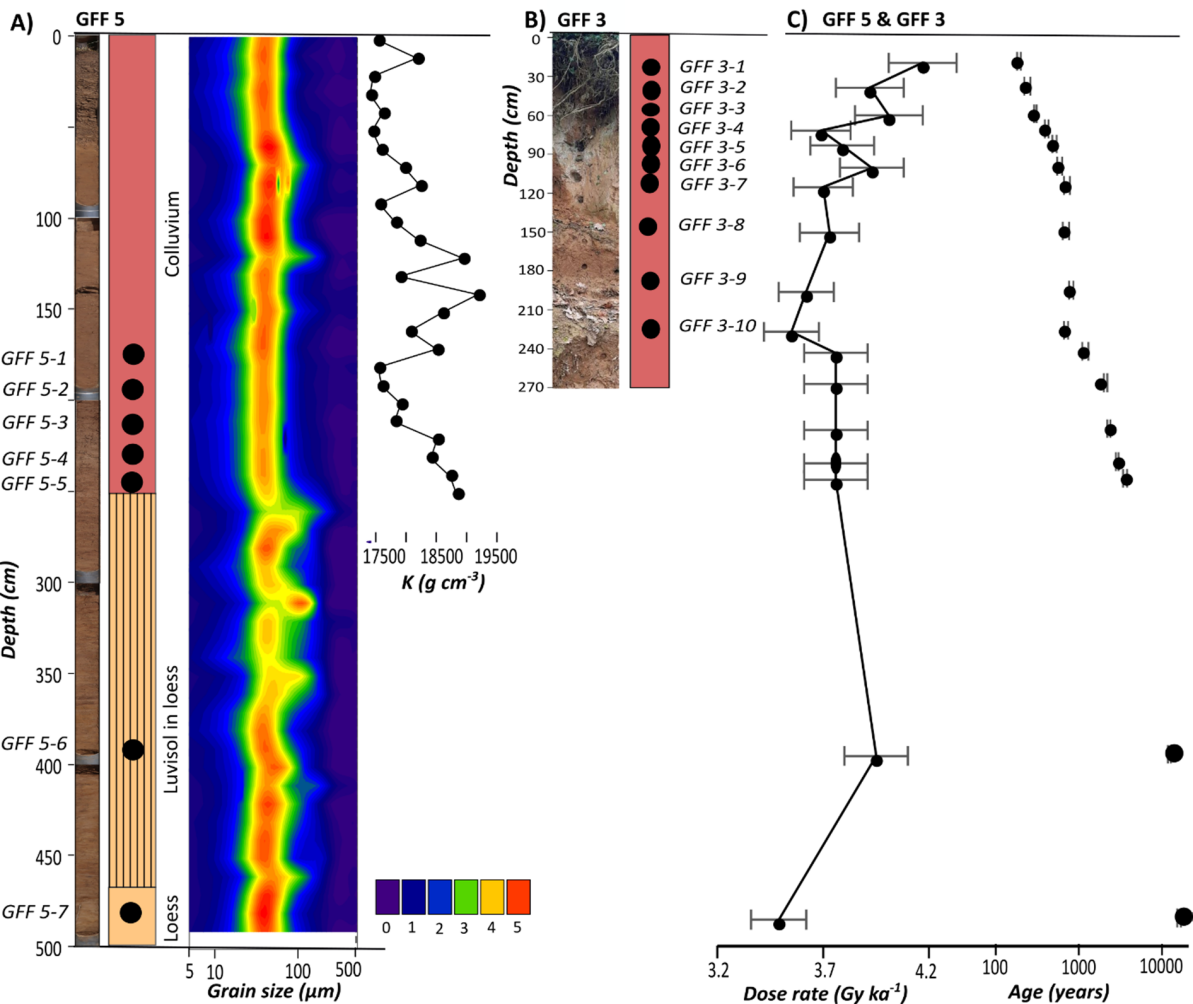
Stratigraphy, sediment composition and luminescence properties for GFF 3 and GFF 5. Please note: GFF 3 (**B**) is scaled differently compared to GFF 5 (**A**) on purpose, to enable a chronological order of the calibration samples in (**C**). Age uncertainties in (**C**) are masked due to the size of the dots.

At GFF, both investigated sections reveal a carbonate-free colluvium, composed of a homogeneous mixture of sand, silt and clay without any clear stratigraphic differentiation (Fig. 3). The material is mainly derived from earlier decalcified soils up slope. In GFF 3, the colluvium has a thickness of at least 270 cm but we did not reach its base during sampling (Fig. 3B). The colluvium in GFF 5 is 250 cm thick and covers carbonate-bearing loess intersected with sandy flood layers, which is still visible at the base of the core. Below the colluvium, between 250 and 470 cm b.s., we found a 220 cm thick, decarbonated Luvisol formed in in-situ loess (Fig. 3A). Sediment analyses conducted for GFF 5 with a resolution of ~ 10 cm reveal overall homogeneous grain sizes with mean values between 19 and 48 μm (27% RSD) for colluvium, paleosoil and loess. Chemical composition was only determined for the colluvium, but shows little variation with respect to elements important for dose rate determination, such as potassium (3% RSD) (Fig. 3A, Tab. S3).

The luminescence properties of the sediments were investigated based on 10 samples from GFF 3 (GFF 3−1 to 3–10) and seven samples from GFF 5 (GFF 5−1 to 5–7), which were dated with conventional luminescence of fine-grain quartz (Fig. 3C). The same samples have been used to calibrate pOSL ages in the following sections. When looking at other factors that have an influence on the pOSL signal besides deposition age, all 17 samples reveal homogeneous properties. Dose rates are in a range of 3.5 to 4.2 Gy ka^− 1^ (5.3% RSD) (Fig. 3C, Tab. S4), and quartz signal sensitivities show relatively small variability between the samples as well (13.6% RSD). Since the low equivalent dose scatter observed for the fine-grain quartz samples is no useful indicator for signal resetting due to strong averaging effects on aliquots containing thousands of grains, we also conducted dating of sand-sized quartz for eight samples from GFF 3. The agreement between fine-grain quartz ages measured on large aliquots and coarse-grain quartz ages measured on aliquots with less than 30 grains (with less averaging between poorly and well bleached grains) for all eight samples within their uncertainties (Fig. S10 in supplement) clearly points to fully reset quartz luminescence signals and supports the suitability of fine-grain quarz for dating. The associated chronology dates the basal loess to 12,300 ± 500 and 16,100 ± 800 years, and the colluvium to 190 ± 10 to 3600 ± 200 years (Fig. 3C). For another four samples from GFF 3 and 5 we also dated the coarse-grain potassium feldspar fraction to evaluate if feldspar signals – which also contribute to bulk pOSL signals – have been bleached as well. While quartz and feldspar ages generally agree with each other for three out of four samples, the feldspar age of sample GFF 5–5 over-estimates the associated quartz age by more than 50% (Fig. S10 in supplement). The effect of incomplete feldspar signal resetting on dating GFF with pOSL is investigated in the following sections.

### Plaggen soil chronology for the Weiner Esch

**Fig. 4:**
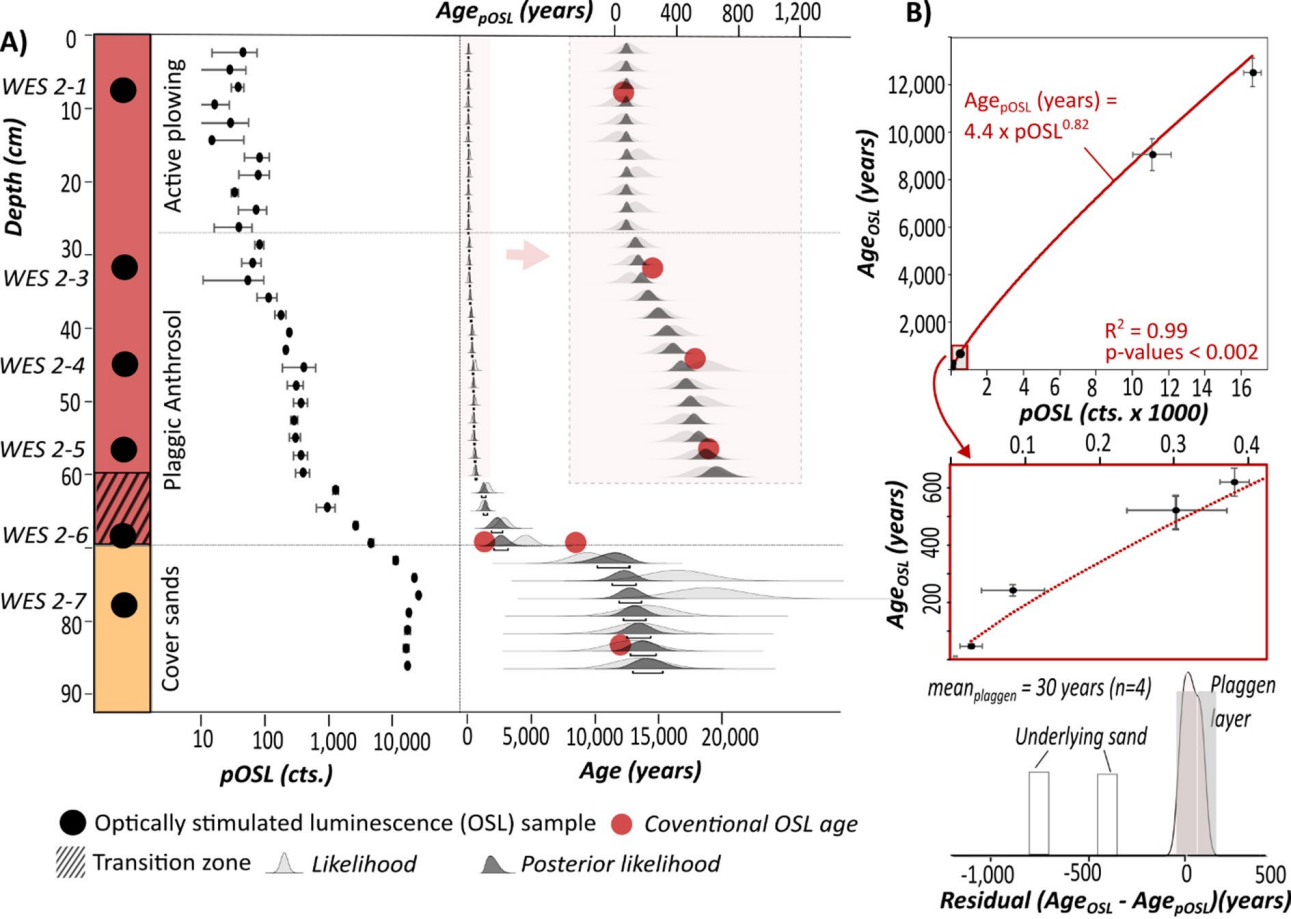
Stratigraphy and luminescence data for plaggen soil from the Weiner Esch. (**A**) High-resolution pOSL data and associated pOSL-based age-depth model against luminescence control ages for sediment core WES 1. Uncertainties of control ages are masked due to the size of the red dots. (**B**) Transfer function between pOSL and deposition age based on OSL ages from core WES 2. Residuals between OSL control ages and pOSL ages are in the range of centuries for the cover sands but only ~ 30 years for the plaggen layer.

To create a high-resolution chronology for WES 1, pOSL was measured every 2 cm. The transfer function to convert pOSL signals into ages is based on the six coarse-grain quartz OSL ages from WES 2 presented in the previous section (Fig. 4). For sample WES 2–6, which is affected by sediment mixing, the cover sand age of 9,100 ± 700 years was used for fitting ages and pOSL with the transfer function, since the pOSL signal is dominated by the large fraction of older grains. As expected from the very homogeneous composition and bleaching of the substrate, control ages and associated pOSL show a strong correlation and negligible residuals for plaggen soil samples when fitted with Eq. (1) (Fig. 2B):

1 $$Ag{e_{pOSL}}\left( {years} \right)\,=\,4.4{\text{ }}x{\text{ }}pOS{L^{0.82}}$$

Combined with Bayesian modeling, the pOSL-based chronology (Fig. 4A) agrees well with the OSL ages of cover sand and the upper 60 cm of the plaggic Anthrosol. Average deposition rates are ~ 0.43 mm/year for this section of the plaggic horizon. However, the pOSL ages are inaccurate for the basal part of the plaggic Anthrosol, where they abruptly rise from 620 ± 90 years at 60 cm to more than 1600 years below. Since the age of the younger grain population in sample WES 2–6 indicates an earliest start of plaggen soil formation at 1300 ± 200 years ago, pOSL ages below 60 cm b.s. appear too old and were excluded from further analysis. This is presumably due to the mixing of older and younger particles, which cannot be resolved within the pOSL measurements.

### Colluvium chronology for Gut Frankenforst

**Fig. 5:**
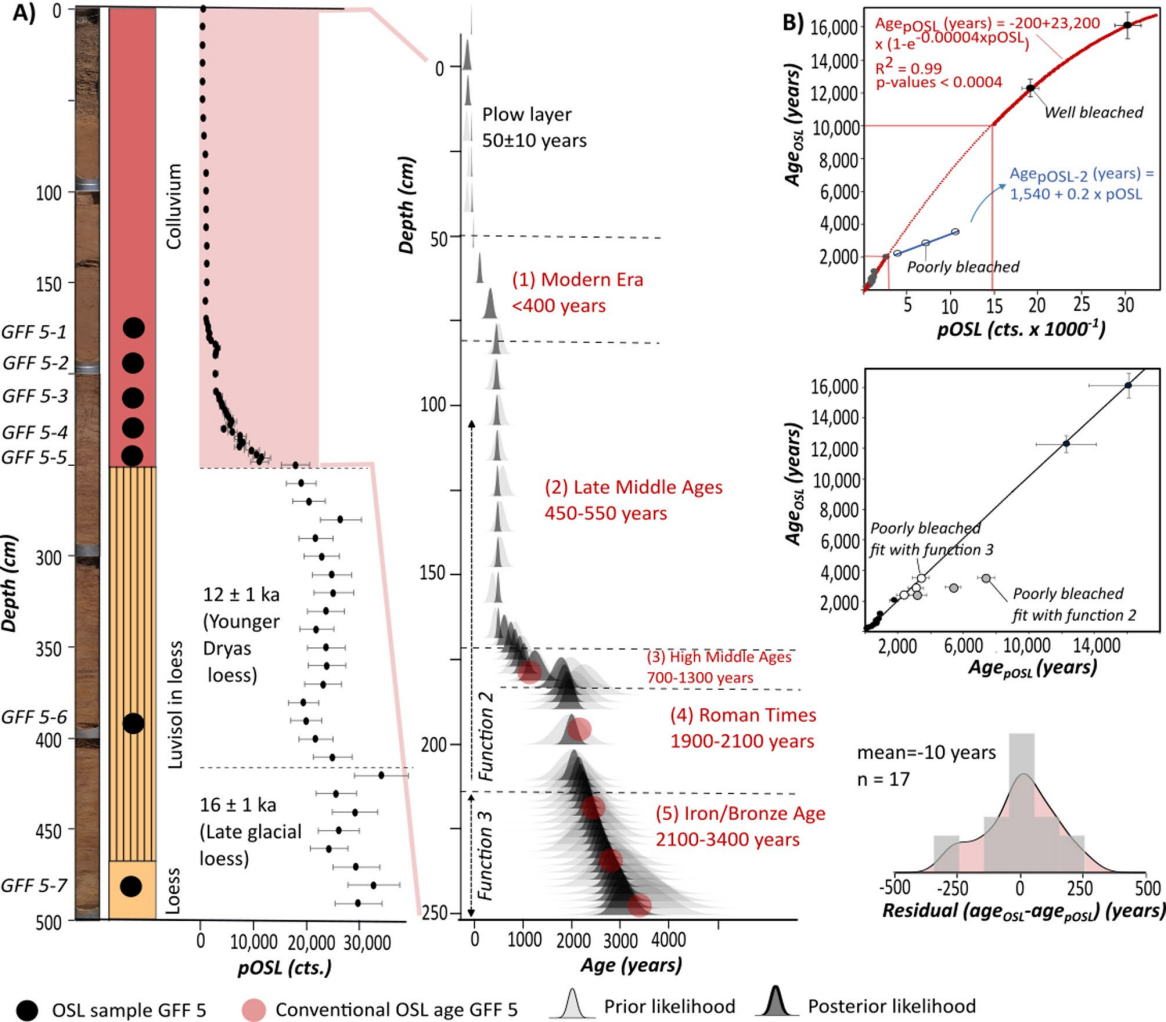
Stratigraphy and luminescence data for colluvium at Gut Frankenforst. (**A**) High-resolution pOSL data and associated age-depth model against OSL control ages for sediment core GFF 5. Uncertainties of control ages are masked due to the size of the red dots. (**B**) Transfer functions between pOSL and deposition age based on OSL ages from GFF 3 and 5. Sections used for different transfer functions are marked in dark red for Eq. (1) and light red for Eq. (2). Residuals between OSL control ages and pOSL ages show a normal distribution around a mean of ~ zero.

To generate a high-resolution colluvium chronology, pOSL was measured every 2–10 cm for GFF 5 (Fig. 5A). For GFF 3, pOSL in high resolution was only measured in the uppermost 90 cm to cover the section masked by active plowing in GFF 5 (Fig. S5 in supplement). Transformation of pOSL into ages is based on the 17 fine-grain quartz OSL ages from GFF 5 and GFF 3 described earlier in this paper. As can be expected from the homogeneous dose rates, signal sensitivities, and luminescence signal resetting, most calibration samples show a strong correlation between pOSL and age (Fig. 5B) when fitted with Eq. (2):

2 $$\:Ag{e}_{pOSL}\left(years\right)=-200+\text{23,200}\text{*}\left(1-{e}^{{-4\text{*}10}^{-5}\text{*}pOSL}\right)$$

However, this is not the case for samples GFF 5−3, 5−4, and 5–5 from the basal part of the colluvium, which have significantly higher pOSL signals than expected from function 2 (Fig. 5B). This is presumably due to the contribution of incompletely bleached feldspar luminescence signals to pOSL, as indicated by the significant age overestimation observed for feldspar sample GFF 5–5 compared to its quartz age (Fig. S10 in supplement). The three poorly bleached samples are better fit by Eq. (3):

3 $$\:Ag{e}_{pOSL}\left(years\right)=0.2*pOSL+\text{1,540}.$$

**Fig. 6:**
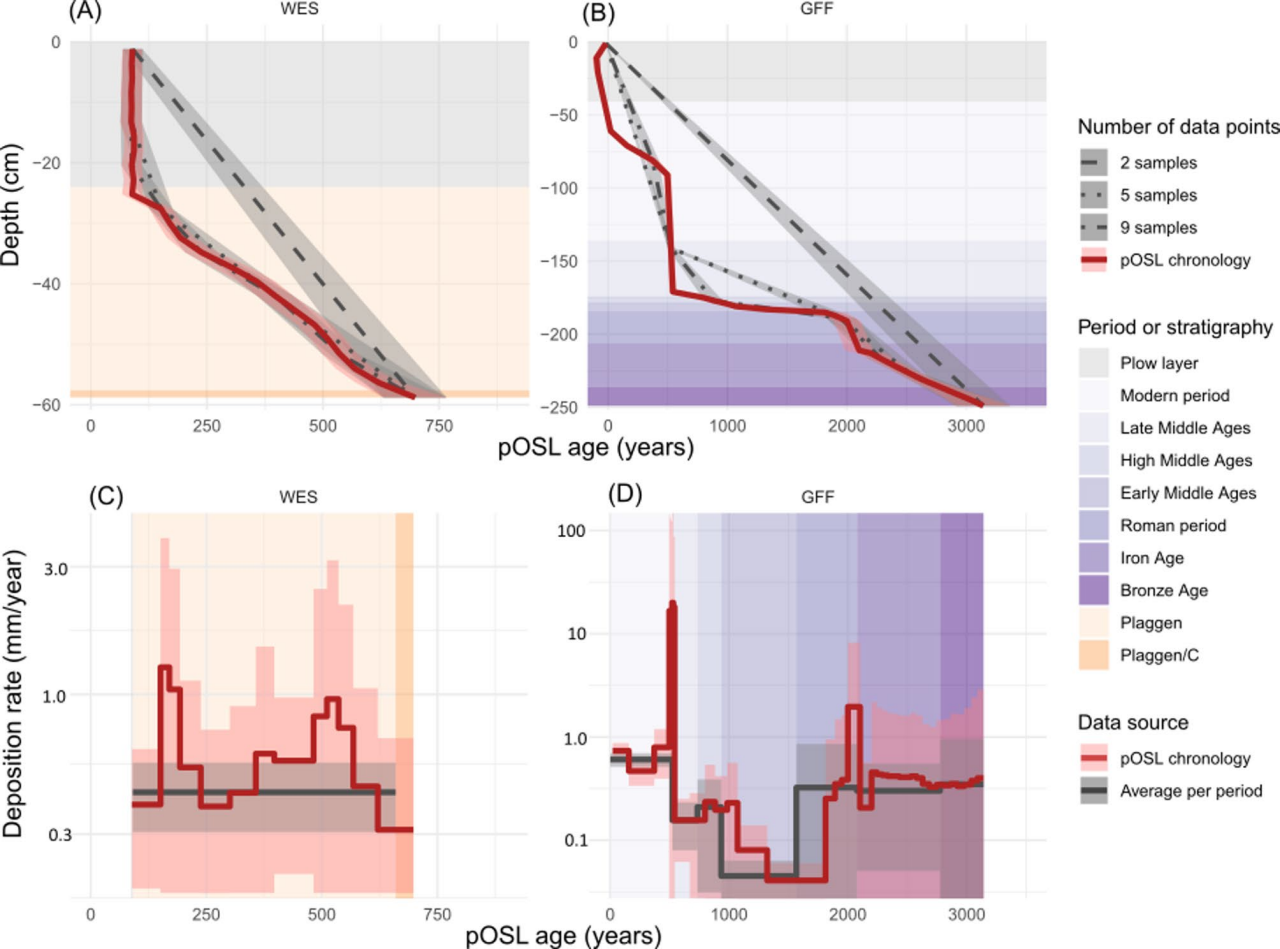
High-resolution pOSL chronologies (red line) for WES (**A**) and GFF (**B**). Grey lines represent age-depth trends when using a limited number of age samples, as would be the case with conventional dating. Ages for constructing these lines were sampled with constant depth intervals from the pOSL age depth curve. (**C**,** D**) High-resolution deposition rates derived from pOSL-chronologies (red line) versus average rates for historic periods (GFF, cf^35^) or stratigraphic units (WES) (black line). The plow layer is left out of the rate calculations. All uncertainties are 1-sigma errors.

We used Eq. (2) to convert pOSL into ages for most of the samples. Only the presumably poorly bleached, basal part of the colluvium at 213–250 cm b.s., which pre-dates the Roman period, was fitted with Eq. (3). After Bayesian modeling, the age-depth model indicates late glacial loess (16,000 ± 1,000 years) under Younger Dryas loess (12,000 ± 1,000 years) in the lower 250 cm (Fig. 5A). These loess ages agree well with previous studies that investigate the Weichselain loess accumulation in the Lower Rhine Area^34^. The new chronology also allows to calculate high-resolution deposition rates for the colluvium. When averaged over historical periods of the Lower Rhine area^35^, they point to rates of 0.3–0.4 mm year^− 1^ during the Bronze to Iron Age, increase to ~ 1.2 mm year^− 1^ in early Roman times, before decreasing to less than 0.1 mm year^− 1^ during the Migration Period. Rates increase to ~ 0.2 mm year^− 1^ during the High to Late Middle Ages, with exceptional rates of ~ 14 mm year^− 1^ at 500–600 years ago (Fig. 6D). Younger soil erosion is masked by tillage in GFF 5, but undisturbed colluvium at GFF 3 (Fig. S5 in supplement) indicates high deposition rates of ~ 1.7 mm year^− 1^ for the past 400 years.

## Discussion

Both substrates provide ideal conditions for establishing high-resolution chronologies based on pOSL, as the luminescence signals are primarily controlled by age. Other factors influencing pOSL, such as grain size, luminescence signal sensitivity, and dose rates, are relatively homogeneous within the samples from each site. Most of the sediments at both sites underwent excellent luminescence signal resetting that we correlate with plowing^36^, either prior to deposition for the tillage-induced colluvium or after deposition for the plaggic soil. Defining one precise value for the number of calibration samples that is need for fitting robust transfer functions is not advisable due to significant site dependency. However, a general suggestion applicable to most settings is at least 5–10 calibration samples including one from the very base and one from the very top of the investigated stratigraphy. This can be guided by pOSL versus depth trends, which helps to differentiate between homogeneous sedimentation requiring less- and heterogeneous sedimentation requiring more calibration samples. As an inherent property of high-resolution chronologies with overlapping age distributions^37^, pOSL-derived deposition rates (Eq. S2) remain somewhat imprecise even after Bayesian modeling (individual deposition rates in Fig. 6C, D). However, the magnitudes are statistically significant and can reveal changes in deposition rates along the profile. Averaging rates over broader time periods (average deposition rates in Fig. 6C, D) helps to constrain uncertainties but at the cost of temporal detail.

For the plaggic soil at WES, our high-resolution pOSL data do not offer additional insights. Due to uniform deposition since the Early Middle Ages, conventional dating provides chronologies comparable to pOSL, even when using just five samples (Fig. 6A, C). Beyond the methodological point of view, the chronology from WES shows average deposition rates of 0.4–0.5 mm year^− 1^. This is slightly lower than reported for most plaggen soils from northern Germany and the Netherlands that rather formed at rates of 1 mm year^− 1^^6^. Also, plaggen soil formation since ~ 700 years CE as calculated for WES starts some hundred years earlier than reported at most sites in literature^6,10^. There are, however, other recent observations from the region that support an early onset of plaggen agriculture^38^.

For the colluvium at GFF, our pOSL chronology offers entirely new insights compared to conventional dating (Fig. 6B, D). The pOSL record suggests that deposition varied by several orders of magnitude over the past 3500 years. Relatively continuous deposition was interrupted by several centuries of virtually no deposition during the Migration period and by short episodes with drastically accelerated deposition during Roman Times and the Late Middle Ages (Fig. 6D). With exceptional rates of ~ 14 mm year^− 1^, the latter episode, in particular, might reflect a catastrophic event, such as the 1342 CE Magdalenian flood^39^, rather than gradual deposition over decades. These deposition rates have to be interpreted with care, since they are based on a single sediment core that – considering potential spatial variability – may not even be representative for local soil erosion dyamics. Nevertheless, the identified succession of phases with accelerated and reduced deposition rates at GFF shows striking parallels to the only yet existing high-resolution soil erosion chronology for the Lower Rhine Area that is based on more than 60 ages for colluvium from the Altdorfer Delle^14^.

The main limitation of the presented approach is the prerequisite that age should be the primary control on pOSL signals^16^. In incompletely bleached and mixed sediments, such as the transition zone at WES and pre-Roman colluvium at GFF, the transfer functions do not perform well because the relationship between pOSL signal and age differs from well-bleached and unmixed sediments. In the basal 10 cm of the plaggic Anthrosol, age over-estimation can be attributed to mixing of plaggen material with cover sands, likely due to low-intensity tillage or natural bioturbation after plaggen formation^12^. The unsuitability of Eq. (2) for the pre-Roman colluvium (213–250 cm) at GFF is likely due to incompletely bleached feldspar luminescence signals (Fig. S10). An explanation for the change in bleaching efficiency could be low-intensity tillage with limited soil mixing as it has been described for the time prior to the Roman period in the area^40^.

Sections where pOSL chronologies deviate from the expected age can either be excluded from further analysis, as we did for WES, or corrected with a secondary transfer function, as we did at GFF. To detect such sections, conventional control ages from the base of the studied substrates are indispensable. However, even if absolute pOSL chronologies remain inaccurate due to a lack of control ages from sections with incomplete luminescence signal resetting, relative changes in the high-resolution pOSL record can guide sample collection for conventional dating, particularly where depositional units cannot be distinguished visually. To verify our results regarding the formation of plaggen soils and the deposition history of colluvium in both study areas, additional cores from various landscape positions need to be dated. The presented pOSL approach has huge potential for this spatial upscaling. Since pOSL transfer functions are substrate- and land-use dependent, they should be valid for similar substrates in a local or even regional context^20^. Thus, with minimal effort, additional sediment cores may be dated using pOSL only, without the need for further conventional dating. While the approach is already more time- and labor-efficient compared to conventional OSL dating for single cores, the benefits increase rapidly when upscaled in local and regional contexts. This sets the foundation for reconstructing not only the temporal but also the spatial patterns of anthropogenic landscape change with unprecedented resolution.

## Conclusions

This study presents, for the first time, chronologies with centimeter-scale resolution for anthropogenic soil substrates based on pOSL measurements, OSL dating, and Bayesian age modeling. The presented workflow offers solutions to challenges related to incomplete pOSL signal resetting and large uncertainties in pOSL-based ages and deposition rates. Despite considerable site dependency, a reasonable estimate for the number of calibration samples per site is between five and ten. The resulting chronologies provide insights into landscape development due to soil erosion and plaggen formation, which would not be revealed by conventional dating approaches with lower temporal resolution. This advantage is particularly significant in settings with heterogeneous deposition histories, such as colluvium, where we identified deposition rates that vary by orders of magnitude with durations ranging from centuries to potentially single events. Since pOSL transfer functions are substrate-dependent, the approach could be scaled in a local to regional context to establish high-resolution spatial and temporal patterns of human-induced landscape evolution without the need for additional OSL dating.

## Methods

### Sample collection

Sediments for luminescence analyses were sampled from a natural outcrop cut by the Forstbach (GFF 3) and cores retrieved with a motor-driven percussion drilling system (Atlas Copco Cobra Pro) equipped with opaque plastic liners in case of GFF 5 and WES. Samples for conventional luminescence dating were collected in steel cylinders from outcrop GFF 3 or taken directly from opaque plastic liners after opening the cores in the luminescence laboratory under red light. pOSL samples were taken either from opaque liners for GFF 5 and WES after opening them in the laboratory, or in a 100-cm long opaque plastic push core at GFF 3. Stratigraphic field descriptions at both sites were supplemented by sedimentological and geochemical investigation in the Physical Geography Laboratory at the University of Cologne.

### Sediment analyses

Grain-size analyses were performed using a Beckman Coulter LS 13 320 laser particle sizer and resulting grain-size distributions were statistically evaluated using the GRADISTAT software^41^. The elemental composition of the samples was determined with energy dispersive X-ray fluorescence (EDXRF) using a Spectro Xepos P EDXRF. Organic carbon contents were measured with a Vario El Cube Eementar C/N analyser.

### Portable OSL reader analyses

To generate high-resolution chronologies for colluvium and plaggen soils, we sampled 2-cm intervals from the cores used for pOSL. All pOSL analyses were carried out under dimmed red-light conditions. Bulk sediment samples were dried at 50 °C and measured with a SUERC portable luminescence reader using successive stimulation by IR LEDs and blue LEDs^16^. POSL signals were calculated by summing the first 10 s of stimulation. Both signals show comparable trends for the samples in this study. However, IRSL signals are derived solely from feldspar minerals, while BSL signals originate from both quartz and feldspar and are, thus, more sensitive to variations of mineral composition between samples. Therefore, we used the IRSL signal for all pOSL analyses in this study. For each sample, the average pOSL signals of 2–3 aliquots were combined with uncertainties estimated based on instrumental error, sample-internal pOSL scatter, and inter-sample scatter of dose rates and luminescence sensitivity, which added up to an additional uncertainty of 15%. The high-resolution pOSL records were then transformed into ages using transfer functions^19^, constructed by correlating pOSL signals with conventional OSL ages determined for the same material.

### Conventional luminescence dating

Conventional OSL samples were pre-processed with standard sieving and chemical proceedures to extract fine-grain (GFF) and coarse-grain (WES and GFF) quartz, as well as coarse grain potassium felspar (GFF). Dose rates were determined via gamma spectrometry using an Ortec PROFILE M-Series GEM Coaxial P-type gamma spectrometer. Equivalent doses of small aliquots with less than 30 grains (coarse-grain quartz and feldspar) and large aliquots with thousands of grains (fine-grain quartz) were measured on Risø TL/OSL DA-20 readers using a standard SAR protocol for quartz^42^ and an adjusted IRSL SAR protocol for feldspar^43^. Burial doses were determined from the associated equivalent dose distributions (*N* = 6 to 17 for fine-grain quartz, *n* = 14 to 38 for coarse-grain quartz, *n* = 11 to 12 for coarse-grain feldspar) based on the central age model (CAM) for completely bleached samples or the minimum age model (MAM) for incompletely bleached samples^44^.

### Bayesian age-depth modelling and deposition rates

We applied Bayesian modeling in OxCal^45^ to generate age-depth models with reduced uncertainties and without age inversions, allowing for the calculation of high-resolution positive deposition rates. We used a Sequence model for the different stratigraphic units, which assumes that ages increase with depth and a Phase model for active plow layers where we expect homogeneous pOSL signals due to mixing^33^. Then, we calculated deposition rates by dividing depth increments by age increments in between two consecutive samples, leaving out samples from the plow layer which would introduce a bias in the deposition rates^36^. The corresponding error was calculated using errors from depth and age increments.

For full details on the methodology see the online supplement material.

## Supplementary Information

Below is the link to the electronic supplementary material.

Supplementary Material 1

## Data Availability

All data that were used in this manuscript are available through the supplementary information associated with this manuscript.
