# Supplementary material for: High-resolution chronologies of anthropogenic soil substrates based on portable luminescence reader data
Source: Sci Rep. 2025 Nov 27;15:42945. doi: 10.1038/s41598-025-29066-3 (PMC12673085; doi:10.1038/s41598-025-29066-3)
Supplement: Supplementary file 1 — Supplementary Material 1 [file 41598_2025_29066_MOESM1_ESM.docx]

**Supplementary material to “High-resolution chronologies of anthropogenic soil substrates based on portable luminescence reader data”**

**Dominik Brill^1*^, Marijn van der Meij^1^, Paula von Lengrießer^1^, Frederike Tschernich^1^, Anja Zander^1^, Stephan Opitz^1^, Tony Reimann^1^**

^1^University of Cologne, Institute of Geography, 50674 Cologne, Germany

* Corresponding author: [brilld@uni-koeln.de](mailto:brilld@uni-koeln.de), ORCID: 0000-0001-8637-4641

***S1. Study areas and site stratigraphy***

The map of plaggen soil distribution (Fig. 1) is based on a compilation of all soil types that contain an anthropogenic, humus-enriched top horizon (plaggen layer) in the digital soil maps of Flanders (Digitale bodemkaart van het Vlaams Gewest 2.0 (1:20.000), VPO, 2017), the Netherlands (BRO Bodemkaart 2023-01 (1:50.000), WENR, 2023) and Germany (Bodenübersichtskarte 2:250.000 (BÜK250) v60, BGR, 2024). The soil maps were compiled in ArcGIS Pro, version 3.4.0. As selection criteria we used: Bodemserie LIKE '%antropogene humus%' (Flanders), first_soilname LIKE '%enkeerd%' And first_soilname NOT LIKE '%kalkhoudend%' (Netherlands) and Buek250_Legende.csv.LE_TXT LIKE '%plaggen%' Or Buek250_Legende.csv.LE_TXT LIKE '%Plaggen%' (Germany).


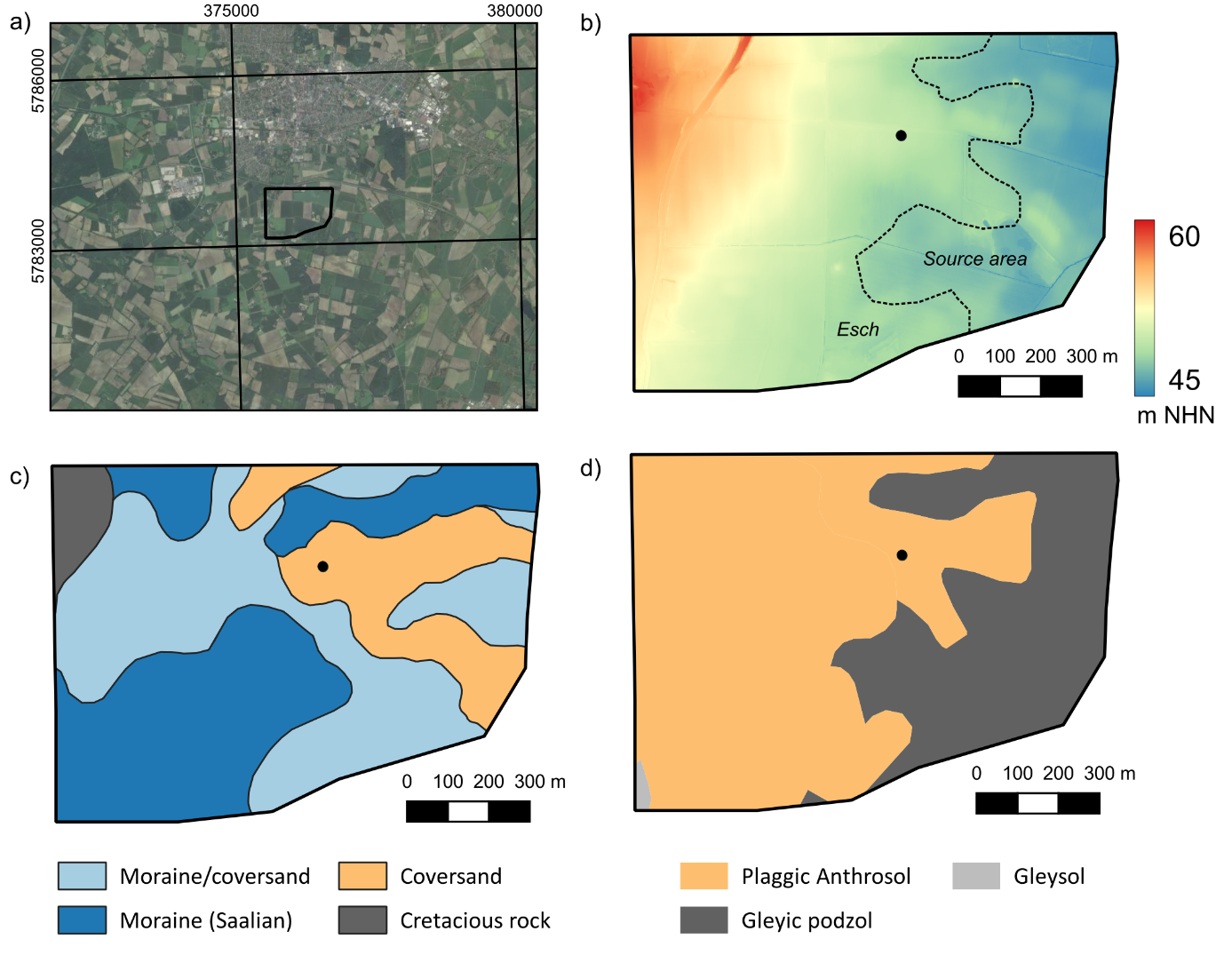


*Fig. S1: Weiner Esch. Location of study site Weiner Esch (WES) south of Ochtrup, Germany (a). Topography (in m above NHN) of the study area (based on* *Geobasis NRW, 2024) (b). Geology of the study area (based on Geologischer Dienst NRW, 2001) (c). Soils of the study area (based on BGR, 2024) (d).*

The Weiner Esch (WES) is located south of Ochtrup, Germany (Fig. S1). The entire area is underlain by Cretacious clay- and limestone covered by glacial till and ground moraine from the penultimate, Saalian (MIS 6 and 8) glaciation (Geologischer Dienst NRW, 2001). The till is partly covered by aeolian cover sands that formed at the end or after the Weichselian (MIS 2) glaciation. These cover sands are porous and poor in nutrients, leading to the formation of gleyic podzols as natural soils, while pseudogleys are the dominant natural soils developed in areas with moraine substrates near the surface (Geologischer Dienst NRW, 2024). Currently, more than 10% of the region and most of the study area is covered by plaggic Anthrosols. The source area of the plaggen material at WES, the Weiner Mark, is located directly to the east of WES. Two 1-m-long sediment cores were retrieved at the eastern rim of the Weiner Esch (Fig. S1), revealing ~70 cm of plaggic anthrosol on top of cover sands.

Gut Frankenforst (GFF) is located in the Pleiser Loess mountains at the southeastern rim of the Lower Rhine Area (Fig. S2). Above Miocene volcanic rocks of the Siebengebirgs volcanism, geological maps indicate several meters of last glacial loess (Geologisches Landesamt NRW, 1978). Along the small streams that drain the catchment (Forstbach and Eichenbach), alluvial sediments have accumulated at the end of the last glacial period and during the Holocene. Samples were collected along a north-facing slope towards the Forstbach, where decalcified Luvisols have formed in the loess (Brinkmann, 2002). Soil erosion led to truncation of these soils at upper and middle slopes, forming calcaric Regosols at locations where the original soil is completely eroded. Colluvic Regosols can be found at lower slopes where colluvium accumulated (Preston, 2001).


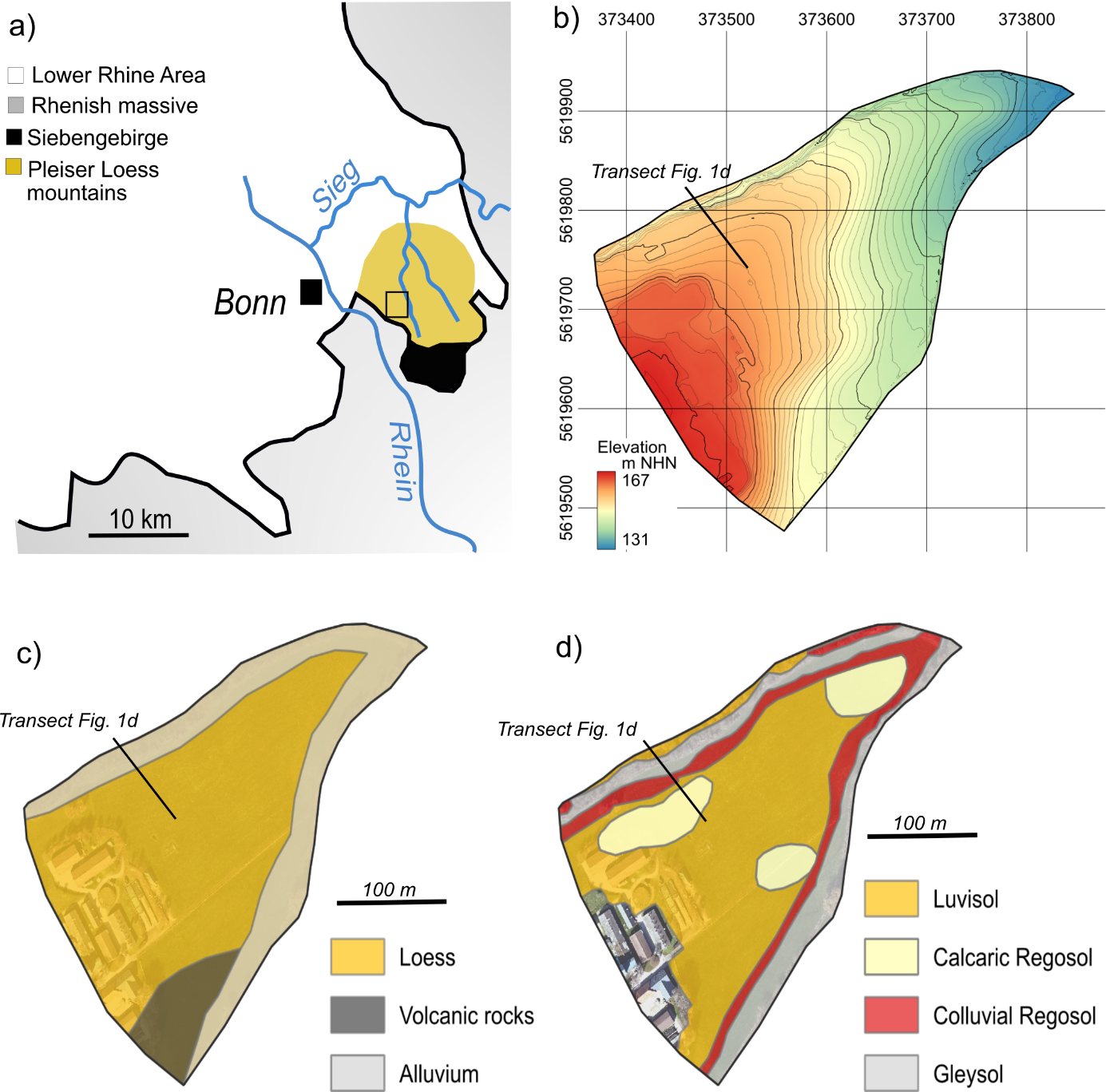


*Fig. S2: Gut Frankenforst. a) Location of study site Gut Frankenforst (GFF) at the southeastern rim of the Lower Rhine Area. b) Topography of the study area (based on* *Geobasis NRW, 2024). c) Geology of the study area (based on Geologisches Landesamt NRW, 1978). d) Soils of the study area (based on Brinkmann, 2002).*

***S2. Field sampling***

Sediments for luminescence analyses at the sampling sites were sampled from a natural outcrop cut by the Forstbach (GFF 3) and cores retrieved with a motor-driven percussion drilling system (Atlas Copco Cobra Pro) equipped with opaque plastic liners in case of GFF 5 (down to 5 m below surface, b.s.) and WES 1 and 2 (down to 1 m b.s.). At GFF 5, overlapping parallel cores were extracted with a vertical offset of 50 cm (i.e. the first core started at the surface, the second at 50 cm below surface). The generated overlap allows to exclude core sections that are affected by collapsed sediments (i.e., typically the upper centimeters to decimeters of each 1-m core segment) from the continuous high-resolution sampling for pOSL. Samples for conventional luminescence dating were collected in steel cylinders from outcrop GFF 3 or taken directly from opaque plastic liners at GFF 5 and WES 2 after opening the cores in the luminescence laboratory under red light. Portable OSL reader (pOSL) samples were taken either from opaque liners for GFF 5 and WES 1 after opening them in the laboratory, or in a 100-cm long opaque plastic push core hammered vertically into the surface next to outcrop GFF 3. The geographical positions of all sample locations are summarized in Table S1.

*Table S1: Geographical information for all sampling locations. Coordinates provided in WGS84 reference system.*

| **Name** | **Latitude (° North)** | **Longitude (° East)** | **Altitude (m above NHN)** |
| --- | --- | --- | --- |
| WES 1 | 52.190176 | 7.188415 | 54.8 |
| WES 2 | 52.190204 | 7.188414 | 54.8 |
| GFF 5 | 50.71617 | 7.207766 | 154.6 |
| GFF 3 | 50.716245 | 7.207567 | 154.0 |

***S3. Sedimentological analyses***

Stratigraphic field descriptions at both sites were supplemented by sedimentological and geochemical investigation with a resolution of ~10 cm in the Physical Geography Laboratory at the University of Cologne. Grain-size analyses were performed using a Beckman Coulter LS 13 320 laser particle sizer. Prior to measurements, samples were dried at 30 °C, sieved to isolate the fraction <2 mm and treated with H_2_O_2_ (15%) at temperatures of 40-60 °C to resolve organic matter and with Na_4_P_2_O_7_ to avoid coagulation. Grain sizes were calculated from laser diffraction patterns using the Fraunhofer model. Resulting grain size distributions were statistically evaluated using the GRADISTAT software (Blott and Pye, 2001). We used statistical values after Folk and Ward (1957) for all further interpretations. The elemental composition of the samples was determined with energy dispersive X-ray fluorescence (EDXRF) using a Spectro Xepos P EDXRF. Prior to measurements, powder samples generated with a mixer mill (Retsch MM 400) and mixed with a Hoechst wax (Cereox binder) to produce hydraulically pressed 2 cm pellets. Organic carbon contents were double measured with a Vario El Cube Eementar C/N analyser. Prior to measurements, powder samples generated with a mixer mill (Retsch MM 400) were placed in tin containers.

The combined stratigraphy of cores WES 1 and WES 2 is composed of basal cover sands between 100 cm and 70 cm b.s. (Fig. S3, Tab. S2). The cover sands are characterized by very low contents of organic carbon (<1%) and leachable elements such as magnesium and phosphorus. The upper 70 cm are formed by a plaggic anthrosol with similar sandy grain sizes but increased contents of organic carbon (>1%) and leachable elements. In particular the elevated phosphorus contents point to fertilization of the plaggen substrate by manure. A mixture of both substrates can be found between 60 cm and 70 cm b.s., where incomplete incorporation of basal sand due to tillage led to the formation of a transitional horizon.


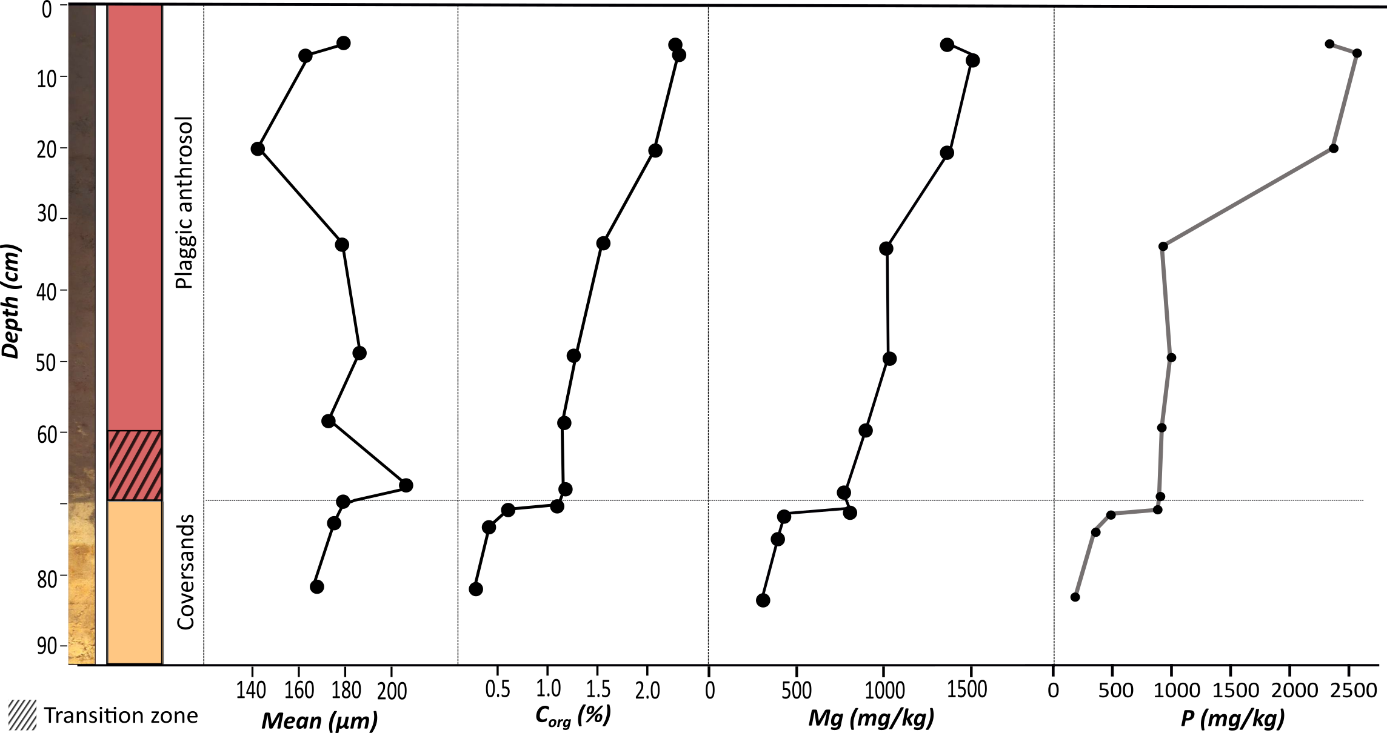


*Fig. S3: Stratigraphy and sedimentology of WES 1 and 2. Mean - mean grain size, C_org_ - organic carbon, Mg – magnesium, P - phosphor.*

*Tab. S2: Sedimentology and geochemistry WES 1 and WES 2.*

| **Sample** | **Depth (cm)** | **Mean**  **(µm)** | **Sorting** | **Skewness** | **Kurtosis** | **C_org_**  **(%)** | **Mg**  **(g/cm³)** | **Mg_Er_ (g/cm³)** | **P**  **(g/cm³)** | **P_Er_ (g/cm³)** |
| --- | --- | --- | --- | --- | --- | --- | --- | --- | --- | --- |
| WES 1-3 | 6 | 177.55 | 2.63 | -0.39 | 2.49 | 2.27 | 1254 | 29 | 2329 | 7 |
| WES 2-1 | 7.3 | 164.4 | 2.71 | -0.41 | 2.57 | 2.27 | 1370 | 30 | 2571 | 7 |
| WES 1-9 | 20.4 | 142.18 | 2.86 | -0.5 | 2.4 | 2.03 | 1245 | 29 | 2367 | 7 |
| WES 2-3 | 33.9 | 178.53 | 2.55 | -0.36 | 2.34 | 1.5 | 883 | 28 | 919 | 4.7 |
| WES 1-21 | 49.2 | 186.16 | 2.43 | -0.36 | 2.46 | 1.25 | 888 | 28 | 988 | 4.8 |
| WES 1-25 | 58.8 | 172.64 | 2.59 | -0.36 | 2.46 | 1.11 | 767 | 27 | 911 | 4.7 |
| WES 1-29 | 68.4 | 207.72 | 2.05 | -0.23 | 1.88 | 1.12 | 635 | 26 | 896 | 4.7 |
| WES 2-6 | 70.2 | 183.83 | 2.09 | -0.26 | 1.97 | 1.07 | 667 | 26 | 877 | 4.6 |
| WES 1-30 | 70.8 | 178.86 | 1.9 | -0.22 | 1.66 | 0.57 | 296 | 22 | 483 | 3.9 |
| WES 1-31 | 73.2 | 175.12 | 1.67 | -0.2 | 1.31 | 0.38 | 270 | 21 | 347 | 3.6 |
| WES 2-7 | 82.3 | 166.02 | 1.58 | -0.15 | 1.19 | 0.22 | 168 | 17 | 181 | 3.2 |

The stratigraphy of 500-cm-long sediment core GFF 5 (Fig. S4, Tab. S3) is composed of basal loess below 475 cm b.s., a silty substrate with >20% carbonate content. Above, a 225 cm thick Luvisol has formed down to 250 cm b.s. with absence of calcium carbonates. The upper 250 cm are composed of colluvial sediments. Grain sizes throughout the entire profile are dominated by silt, with occasional sandier sections. The colluvium has a more homogeneous, slightly finer texture. Within the colluvium mean grain size rises from values of 20-30 µm to peak levels of ~40 µm between ~90 cm and 150 cm. In the same section, Fe/Si ratios (and other geochemical markers for weathering) reach minimum values within the colluvium. The uppermost ~30 cm have been mixed by tillage and form a plow layer with darker colour and slightly higher organic carbon contents.

*Tab. S3: Sedimentology and geochemistry GFF 5.*

| ***Sample*** | ***Depth*** | ***Mean*** | ***Sorting*** | ***Mg  (g/cm³)*** | ***Mg_Er_  (g/cm³)*** | ***Si  (g/cm³)*** | ***Si_Er_  (g/cm³)*** | ***P  (g/cm³)*** | ***P_Er_  (g/cm³)*** | ***Fe  (g/cm³)*** | ***Fe_Er_  (g/cm³)*** |
| --- | --- | --- | --- | --- | --- | --- | --- | --- | --- | --- | --- |
| GFF 5-S1 | 1 | 26.5 | 4.3 | 6910 | 21 | 270950 | 3889 | 1373 | 14 | 22625 | 205 |
| GFF 5-S2 | 11 | 20.0 | 4.1 | 7412 | 29 | 278600 | 566 | 1218 | 8 | 25065 | 49 |
| GFF 5-S3 | 21 | 22.6 | 4.2 | 7791 | 205 | 286850 | 354 | 1252 | 69 | 25830 | 28 |
| GFF 5-S4 | 31 | 19.0 | 3.9 | 7573 | 52 | 280250 | 2758 | 1176 | 42 | 25940 | 113 |
| GFF 5-S5 | 41 | 22.6 | 4.3 | 7478 | 43 | 284000 | 424 | 1104 | 17 | 24980 | 71 |
| GFF 5-S6 | 51 | 24.4 | 4.1 | 7800 | 171 | 281400 | 566 | 1148 | 18 | 25290 | 71 |
| GFF 5-S7 | 61 | 31.7 | 3.4 | 7289 | 117 | 291000 | 566 | 614 | 12 | 23645 | 7 |
| GFF 5-S8 | 71 | 43.8 | 3.7 | 7155 | 10 | 300300 | 0 | 670 | 3 | 22565 | 7 |
| GFF 5-S9 | 81 | 37.5 | 3.5 | 6093 | 59 | 301100 | 424 | 460 | 3 | 21050 | 14 |
| GFF 5-S10 | 91 | 35.8 | 4.0 | 5999 | 156 | 306450 | 3323 | 502 | 40 | 20150 | 42 |
| GFF 5-S11 | 101 | 24.5 | 3.6 | 5781 | 197 | 308500 | 4950 | 492 | 35 | 20345 | 106 |
| GFF 5-S12 | 111 | 27.3 | 3.6 | 6064 | 26 | 311950 | 1485 | 501 | 4 | 20740 | 28 |
| GFF 5-S13 | 121 | 41.9 | 3.7 | 5888 | 48 | 306150 | 71 | 511 | 13 | 20250 | 14 |
| GFF 5-S14 | 131 | 23.8 | 4.1 | 6302 | 135 | 303900 | 566 | 567 | 13 | 20570 | 28 |
| GFF 5-S15 | 141 | 27.2 | 4.7 | 6362 | 38 | 308900 | 566 | 549 | 9 | 22090 | 28 |
| GFF 5-S16 | 151 | 33.0 | 4.3 | 6693 | 25 | 297500 | 1273 | 673 | 24 | 23890 | 71 |
| GFF 5-S17 | 161 | 22.3 | 4.2 | 6840 | 29 | 297450 | 354 | 596 | 5 | 22900 | 42 |
| GFF 5-S18 | 171 | 18.7 | 4.0 | 6912 | 61 | 295650 | 71 | 588 | 16 | 23555 | 7 |
| GFF 5-S19 | 181 | 23.0 | 4.9 | 6371 | 110 | 289850 | 354 | 649 | 1 | 24200 | 28 |
| GFF 5-S20 | 191 | 19.3 | 4.7 | 5878 | 4 | 291850 | 495 | 653 | 8 | 22495 | 21 |
| GFF 5-S21 | 201 | 20.3 | 4.7 | 6386 | 26 | 299250 | 354 | 665 | 5 | 22830 | 0 |
| GFF 5-S22 | 211 | 17.5 | 4.4 | 6281 | 90 | 293900 | 990 | 780 | 11 | 23415 | 64 |
| GFF 5-S23 | 221 | 18.6 | 4.6 | 5872 | 29 | 296150 | 2758 | 638 | 11 | 22030 | 99 |
| GFF 5-S24 | 231 | 20.0 | 4.5 | 5383 | 37 | 304650 | 354 | 569 | 10 | 20120 | 0 |
| GFF 5-S25 | 241 | 22.3 | 4.4 | 5524 | 51 | 312300 | 1556 | 454 | 18 | 19580 | 14 |
| GFF 5-S26 | 251 | 30.7 | 4.4 | - | - | - | - | - | - | - | - |
| GFF 5-S27 | 261 | 47.6 | 4.2 | - | - | - | - | - | - | - | - |
| GFF 5-S28 | 271 | 38.7 | 3.7 | - | - | - | - | - | - | - | - |
| GFF 5-S29 | 281 | 31.1 | 3.7 | - | - | - | - | - | - | - | - |
| GFF 5-S30 | 291 | 25.1 | 4.3 | - | - | - | - | - | - | - | - |
| GFF 5-S31 | 301 | 31.2 | 4.1 | - | - | - | - | - | - | - | - |
| GFF 5-S32 | 311 | 43.3 | 3.7 | - | - | - | - | - | - | - | - |
| GFF 5-S33 | 321 | 37.0 | 4.2 | - | - | - | - | - | - | - | - |
| GFF 5-S34 | 331 | 31.0 | 4.2 | - | - | - | - | - | - | - | - |
| GFF 5-S35 | 341 | 28.1 | 4.2 | - | - | - | - | - | - | - | - |
| GFF 5-S36 | 351 | 41.1 | 3.8 | - | - | - | - | - | - | - | - |
| GFF 5-S37 | 361 | 45.8 | 4.2 | - | - | - | - | - | - | - | - |
| GFF 5-S38 | 371 | 25.9 | 4.0 | - | - | - | - | - | - | - | - |
| GFF 5-S39 | 381 | 21.3 | 4.0 | - | - | - | - | - | - | - | - |
| GFF 5-S40 | 391 | 28.7 | 3.9 | - | - | - | - | - | - | - | - |
| GFF 5-S41 | 401 | 31.1 | 3.7 | - | - | - | - | - | - | - | - |
| GFF 5-S42 | 411 | 41.4 | 4.1 | - | - | - | - | - | - | - | - |
| GFF 5-S43 | 421 | 35.0 | 3.4 | - | - | - | - | - | - | - | - |
| GFF 5-S44 | 431 | 32.9 | 3.8 | - | - | - | - | - | - | - | - |
| GFF 5-S45 | 441 | 32.8 | 3.7 | - | - | - | - | - | - | - | - |
| GFF 5-S46 | 451 | 33.8 | 3.6 | - | - | - | - | - | - | - | - |
| GFF 5-S47 | 461 | 38.5 | 3.7 | - | - | - | - | - | - | - | - |
| GFF 5-S48 | 471 | 33.9 | 3.4 | - | - | - | - | - | - | - | - |
| GFF 5-S49 | 481 | 30.8 | 3.5 | - | - | - | - | - | - | - | - |
| GFF 5-S50 | 491 | 29.2 | 3.5 | - | - | - | - | - | - | - | - |

The stratigraphy of 270-cm-thick sediment outcrop GFF 3 is formed completely by colluvium (Fig. S5). The sediments do not show any significant variations in granulometry or geochemistry, nor do they show any macroscopic stratigraphic boundaries. Different from nearby sediment core GFF 5, the uppermost decimetres of GFF 3 are not affected by tillage. Instead of a plow layer, the top of the sequence is formed by a thin humic topsoil. In addition to 10 full luminescence dating samples spread over the profile for age calibration, the uppermost 90 cm were sampled for pOSL with a resolution of one sample every ~3 cm to investigate the most recent colluvium chronology without any interference by plowing. Between the surface and 90 cm depth, pOSL-based ages rise more or less continuously to ~400 years.


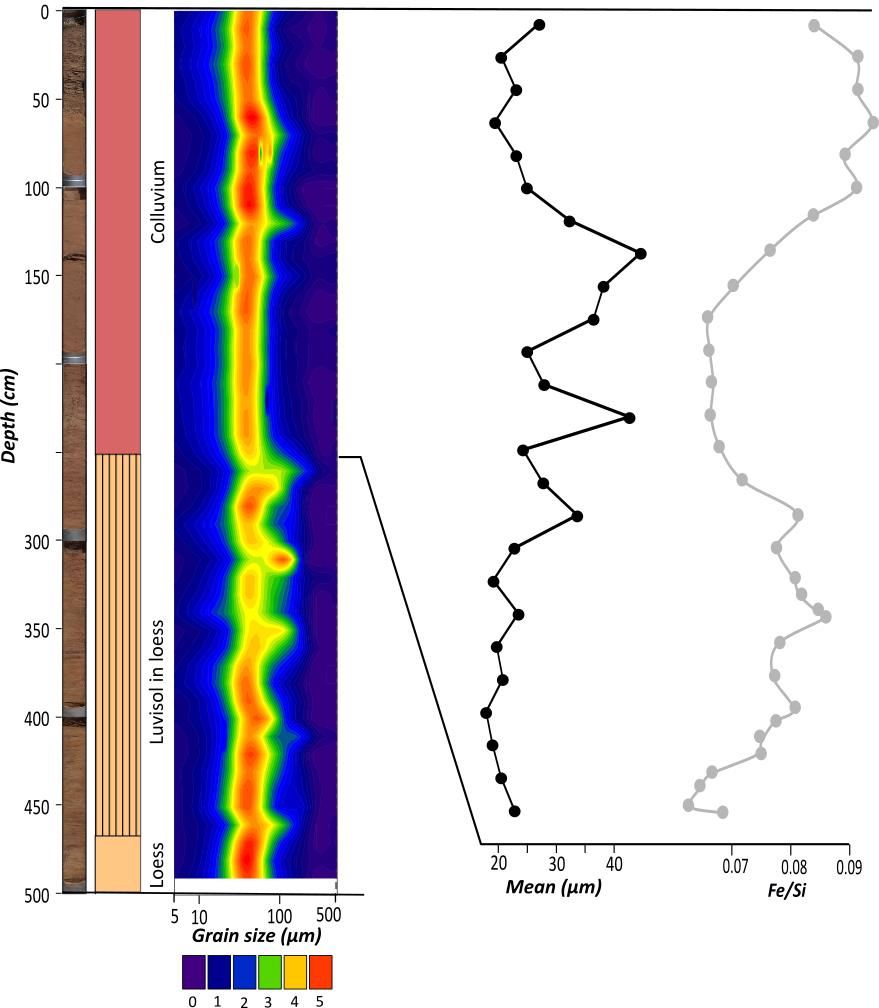


*Fig. S4: Stratigraphy and sedimentology GFF 5. Grain-size heat map (values in percentage) generated using SigmaPlot 15.0. Mean – mean grain size, Fe/Si – ratio between XRF-derived iron and silica values.*


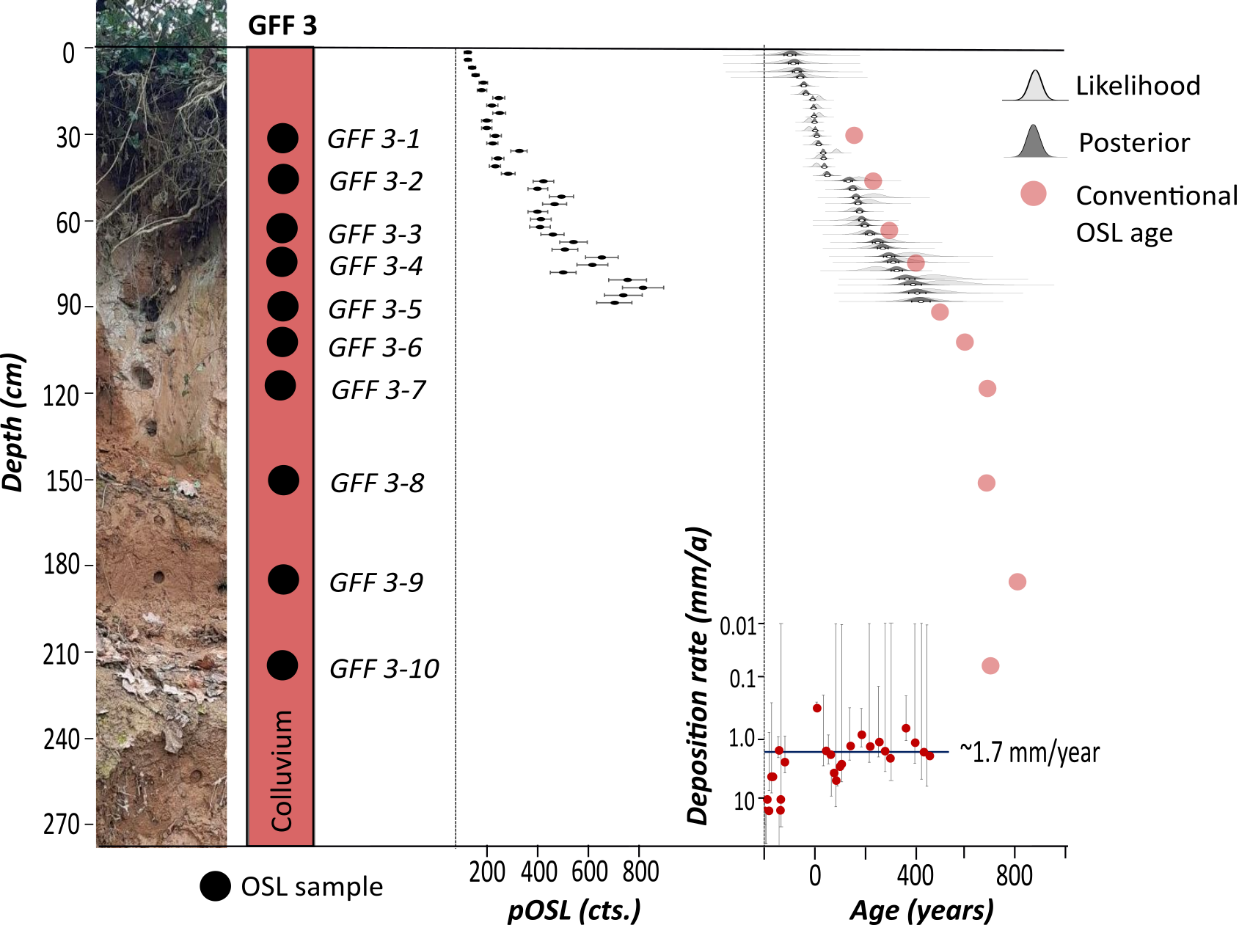


*Fig. S5: Stratigraphy, sedimentology and chronology of outcrop GFF 3.*

***S4. Conventional luminescence dating***

For six sediment samples from WES 2 and 17 sediment samples from GFF 3 and GFF 5, conventional quartz OSL ages were determined at the Cologne Luminescence Laboratory (CLL). Luminescence dating for the WES samples was conducted using coarse-grain quartz (100-200 µm). For the GFF samples, fine-grain quartz (4-11 µm) was used. Ten of the fine-grain GFF samples were cross-checked with coarse-grain (150-250 µm) quartz ages, and four of them with coarse-grain (100-200 µm) feldspar IRSL ages.

***S4.1 Sample preparation***

Sediment cores were split and sampled under dimmed red-light conditions. Sample preparation for coarse-grain quartz included dry sieving to isolate sand-sized grains, treatment with HCl (10%) to remove carbonates, with H_2_O_2_ (10%) to remove organic matter, with Na_2_C_2_O_4_ (0.01 N) to remove clay aggregates and coatings, and density separation with sodium polytungstate (2.62 g cm^-^³ < quartz < 2.68 g cm^-^³) to isolate the quartz fraction. Afterwards, a final HF etch (40% for 40 minutes) followed by an HCl (10%) wash to remove remaining feldspars, was used to remove the alpha-radiation affected rim of the quartz grains and any fluorides that may have formed during the etching process. Preparation of fine-grain quartz followed the same geochemical treatments but used separation of the 4-11 µm fraction with suspension settling (fraction <11 µm) and a centrifuge (fraction >4 µm) and final etching with H_2_SiF_6_ for 7 days to remove anything but quartz. Coarse-grain feldspar extracts were isolated with the same steps as described for coarse-grain quartz, except from using different densities for mineral separation (feldspar < 2.58 g cm^-^³) and without any HF treatment.

***S4.2 Dose rate determination***

Dose rates for all grain-size and mineral fractions are based on radionuclide contents of the samples derived with high-resolution gamma spectrometry with an Ortec PROFILE M-Series GEM Coaxial P-type gamma spectrometer and the conversion factors of Guérin et al. (2011). Water contents were determined by measuring the water loss after drying the samples at 50 °C. Internal dose rates for feldspar extracts are based on empirical potassium contents of 10±2% (Smedley et al., 2012). The alpha contribution is calculated with a-values of 0.04±0.01 for quartz (Lai et al., 2008) and 0.07±0.02 for feldspar (Kreutzer et al., 2014). Cosmic dose rates were estimated on the basis of geographic location, depth below surface and a sediment density of 1.8±0.1 g cm^-^³ following Prescott and Hutton (1994). Calculation of total dose rates was based on the online calculator DRAC (Durcan et al., 2015). Data related to dose rate calculation are summarized in Table S4 for all samples.

*Tab. S4: Summary of dose rate data for all quartz samples used in this study.*

|  |  |  |  |  |  |  |  |
| --- | --- | --- | --- | --- | --- | --- | --- |
| **Sample** | **Lab-ID** | **Depth (cm)** | **U (ppm)** | **Th (ppm)** | **K (%)** | **Water (%)** | **Dose rate (Gy ka^-1^)** |
| WES 2-1 | C-L5658 | 7 | 0.75±0.06 | 2.5±0.19 | 0.8±0.02 | 15±5 | 1.22±0.05 |
| WES 2-3 | C-L5660 | 34 | 0.75±0.06 | 2.4±0.19 | 0.8±0.02 | 15±5 | 1.24±0.05 |
| WES 2-4 | C-L5661 | 45 | 0.74±0.06 | 2.2±0.18 | 0.8±0.02 | 15±5 | 1.18±0.05 |
| WES 2-5 | C-L5662 | 58 | 0.76±0.06 | 2.2±0.17 | 0.8±0.02 | 15±5 | 1.14±0.05 |
| WES 2-6 | C-L5663 | 70 | 0.68±0.05 | 1.8±0.14 | 0.8±0.02 | 15±5 | 1.14±0.05 |
| WES 2-7 | C-L5664 | 82 | 0.48±0.04 | 1.5±0.12 | 0.9±0.02 | 15±5 | 1.10±0.05 |
| GFF 3-1 | C-L5130 | 27 | 3.37±0.23 | 12.0±0.83 | 1.8±0.05 | 15±5 | 4.17±0.16 |
| GFF 3-2 | C-L5131 | 45 | 3.36±0.23 | 12.2±0.77 | 1.9±0.05 | 15±5 | 3.92±0.16 |
| GFF 3-3 | C-L5132 | 65 | 3.47±0.24 | 12.5±0.87 | 1.9±0.05 | 15±5 | 4.01±0.16 |
| GFF 3-4 | C-L5133 | 76 | 3.15±0.21 | 11.9±0.82 | 1.9±0.05 | 15±5 | 3.69±0.14 |
| GFF 3-5 | C-L5134 | 87 | 3.60±0.24 | 12.4±0.87 | 1.9±0.05 | 15±5 | 3.79±0.15 |
| GFF 3-6 | C-L5135 | 103 | 3.31±0.23 | 12.4±0.87 | 1.8±0.05 | 15±5 | 3.93±0.15 |
| GFF 3-7 | C-L5136 | 117 | 3.17±0.22 | 12.0±0.78 | 1.8±0.05 | 15±5 | 3.7±0.14 |
| GFF 3-8 | C-L5137 | 150 | 3.38±0.23 | 12.4±0.87 | 1.8±0.05 | 15±5 | 3.73±0.14 |
| GFF 3-9 | C-L5138 | 193 | 3.19±0.22 | 12.1±0.84 | 1.8±0.05 | 15±5 | 3.62±0.13 |
| GFF 3-10 | C-L5139 | 222 | 3.45±0.23 | 12.8±0.89 | 1.9±0.05 | 15±5 | 3.55±0.13 |
| GFF 5-1 | C-L5757 | 178 | 3.16±0.21 | 12.5±0.87 | 2.1±0.05 | 15±5 | 3.76±0.15 |
| GFF 5-2 | C-L5758 | 195 | 3.16±0.21 | 12.5±0.87 | 2.1±0.05 | 15±5 | 3.76±0.15 |
| GFF 5-3 | C-L5991 | 220 | 3.16±0.21 | 12.5±0.87 | 2.1±0.05 | 15±5 | 3.76±0.15 |
| GFF 5-4 | C-L5759 | 238 | 3.16±0.21 | 12.5±0.87 | 2.1±0.05 | 15±5 | 3.76±0.15 |
| GFF 5-5 | C-L5760 | 247 | 3.16±0.21 | 12.5±0.87 | 2.1±0.05 | 15±5 | 3.76±0.15 |
| GFF 5-6 | C-L5708 | 397 | 3.16±0.21 | 12.4±0.87 | 2.1±0.05 | 15±5 | 3.95±0.15 |
| GFF 5-7 | C-L5709 | 486 | 3.17±0.22 | 11.5±0.81 | 1.7±0.04 | 15±5 | 3.49±0.13 |

***S4.3 Dose determination***

For burial dose determination, quartz and potassium feldspar extracts were fixed on steel discs in patches of 1 mm (coarse grains GFF) and 2 mm (coarse grains WES) using silicon oil. For the fine-grain samples 1 mg per disc were pipetted on steel discs to create monolayers with 9.8 mm diameter. All discs were measured on Risø TL/OSL DA-20 readers equipped with ^90^Sr/^90^Y beta irradiation sources delivering ~0.1 Gy/s at the sample position. Luminescence signals were stimulated with blue LEDs (~470 nm) at 125 °C (quartz) and infrared LEDs (~870 nm) at 50 °C (feldspar), and detected through 7.5 mm Hoya U340 (quartz) or 410 nm interference filters (feldspar). All quartz measurements followed a st­­­andard SAR protocol (Murray and Wintle, 2000) with preheat at 200 °C (GFF) or 180 °C (WES) for 10 s, cutheat at temperatures 20 °C below the preheat and a hotbleach at 190 °C (only for WES) (Fig. S6). Aliquots were rejected from further analyses if their recycling ratio and depletion ratio deviated by more than 15% from unity, and if the test dose error exceeded 10%.

| ***a) Quartz OSL GFF*** | |  |  | ***c) Feldspar IRSL GFF*** | |  |
| --- | --- | --- | --- | --- | --- | --- |
| **Step** | **Treatment** | **Signal** |  | **Step** | **Treatment** | **Signal** |
| 1 | Preheat (200 °C for 10 s) |  |  | 1 | Preheat (180 °C for 60 s) |  |
| 2 | Blue LEDs (40 s @ 125 °C) | Lx (OSL) |  | 2 | IRSL LEDs (300 s @ 50 °C) | Lx (IRSL) |
| 3 | Test dose |  |  | 3 | Test dose |  |
| 4 | Cutheat (180 °C) |  |  | 4 | Preheat (180 °C for 60 s) |  |
| 5 | Blue LEDs (40 s @ 125 °C) | Tx (OSL) |  | 5 | IRSL LEDs (300 s @ 50 °C) | Tx (IRSL) |
| 6  7 | Dose (R1-R4, R0, RR)  Depletion ratio |  |  | 6 | Dose (R1-R4, R0, RR) |  |
| 8 | Return to step 1 |  |  | 7 | Return to step 1 |  |
|  |  |  |  |  |  |  |
| ***b) Quartz OSL WES*** | |  |  |  |  |  |
| **Step** | **Treatment** | **Signal** |  |  |  |  |
| 1 | Preheat (180 °C for 10 s) |  |  |  |  |  |
| 2 | Blue LEDs (40 s @ 125 °C) | Lx (OSL) |  |  |  |  |
| 3 | Test dose |  |  |  |  |  |
| 4 | Cutheat (160 °C) |  |  |  |  |  |
| 5 | Blue LEDs (40 s @ 125 °C) | Tx (OSL) |  |  |  |  |
| 6 | Hotbleach (40s @ 190 °C) |  |  | *R1-x = regeneration doses* | | |
| 7  8 | Dose (R1-R3, R0, RR)  Depletion ratio |  |  | *RR = recycling dose* | | |
| 9 | Return to step 1 |  |  | *R0 = zero dose (recuperation)* | | |

*Fig. S6: Measurement protocols for quartz and feldspar luminescence samples from GFF and WES.*

The appropriateness of thermal pretreatment for quartz was tested with preheat-plateau tests for selected samples from each site that indicate equivalent doses independent of preheat temperatures between 160 °C and 260 °C for all samples (Fig. S7). The protocols were evaluated by means of dose-recovery tests that demonstrate adequate reproducibility of laboratory doses in the range of the natural dose for all samples (dose recovery ratios of 0.93-1.04; Fig. S8). For feldspar samples an adapted SAR protocol (Wallinga et al., 2000) with preheat at 180 °C for 60 s was applied and evaluated using dose-recovery tests (dose-recovery ratios of 0.97-1.04) and residual-dose experiments (residual doses <0.2 Gy) both after signal resetting in a Dr. Hönle Sol2 solar simulator for 24 hours.


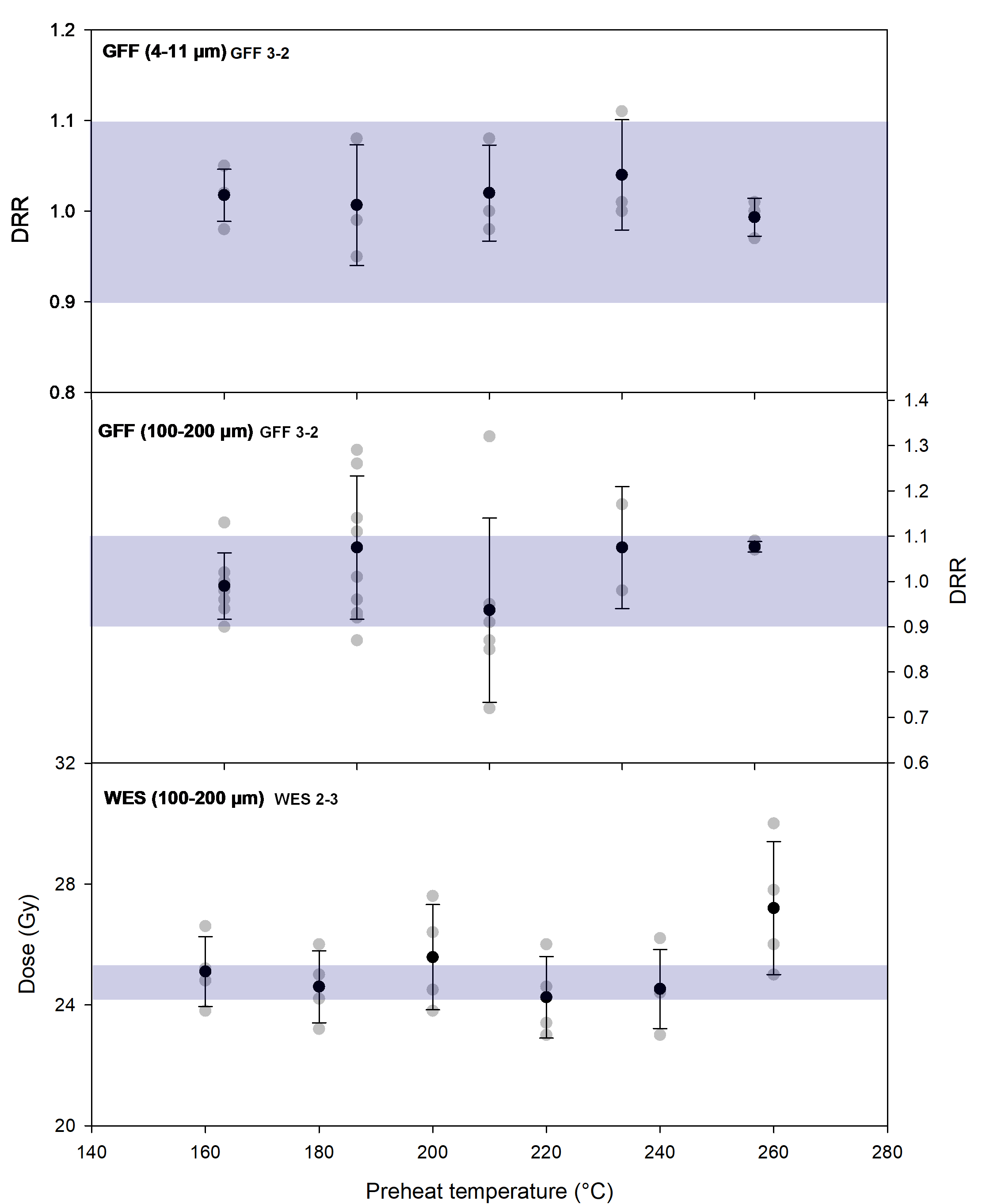


*Fig. S7: Preheat tests for quartz samples from GFF and WES. Dose-recovery-preheat-plateau tests with a laboratory dose of ~5 Gy were performed for a representative sample (i.e. GFF 3-2) of fine-grain and coarse-grain quartz from GFF. For a representative sample from WES (WES 2-3), a simple preheat-plateau test with natural signals was conducted. Individual data of 3-4 aliquts per temperature are shown in grey, mean values in black. Preheat plateau for WES and acceptable dose recovery ratios (DRR) are marked by shaded areas.*


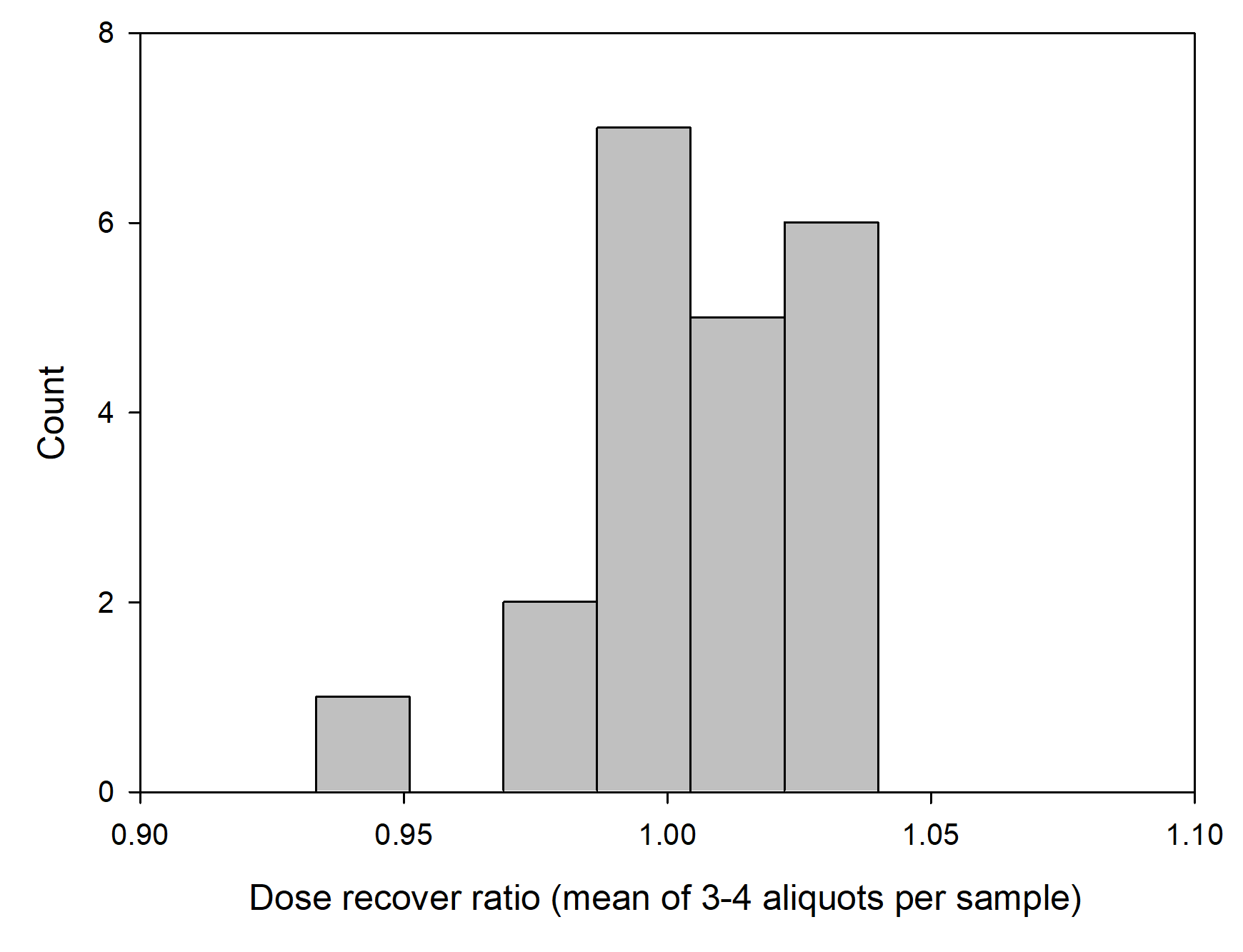


*Fig. S8: Dose-recovery ratios of all samples (GFF and WES) analysed in this study (N = 23). The mean values plotted here are based on 3-4 aliquots per sample.*

***S4.5 Burial dose and age calculations***

Due to negligible equivalent dose scatter for most of the samples (Fig. S9, Tab. S5) and good bleaching before and after deposition due to plowing activity, the central age model (CAM, Galbraith et al., 1999) was used to calculate burial doses for both minerals (quartz and feldspar) and grain-size fractions (silt and sand) of most samples. Only for coarse-grain quartz sample WES 2-6, sampled from the transition zone between plaggic horizon and underlying coversand, the minimum age model (MAM, Galbraith et al., 1999) was additionally applied to extract the dose of the younger population corresponding to the plaggen soil substrate (Fig. S9b).


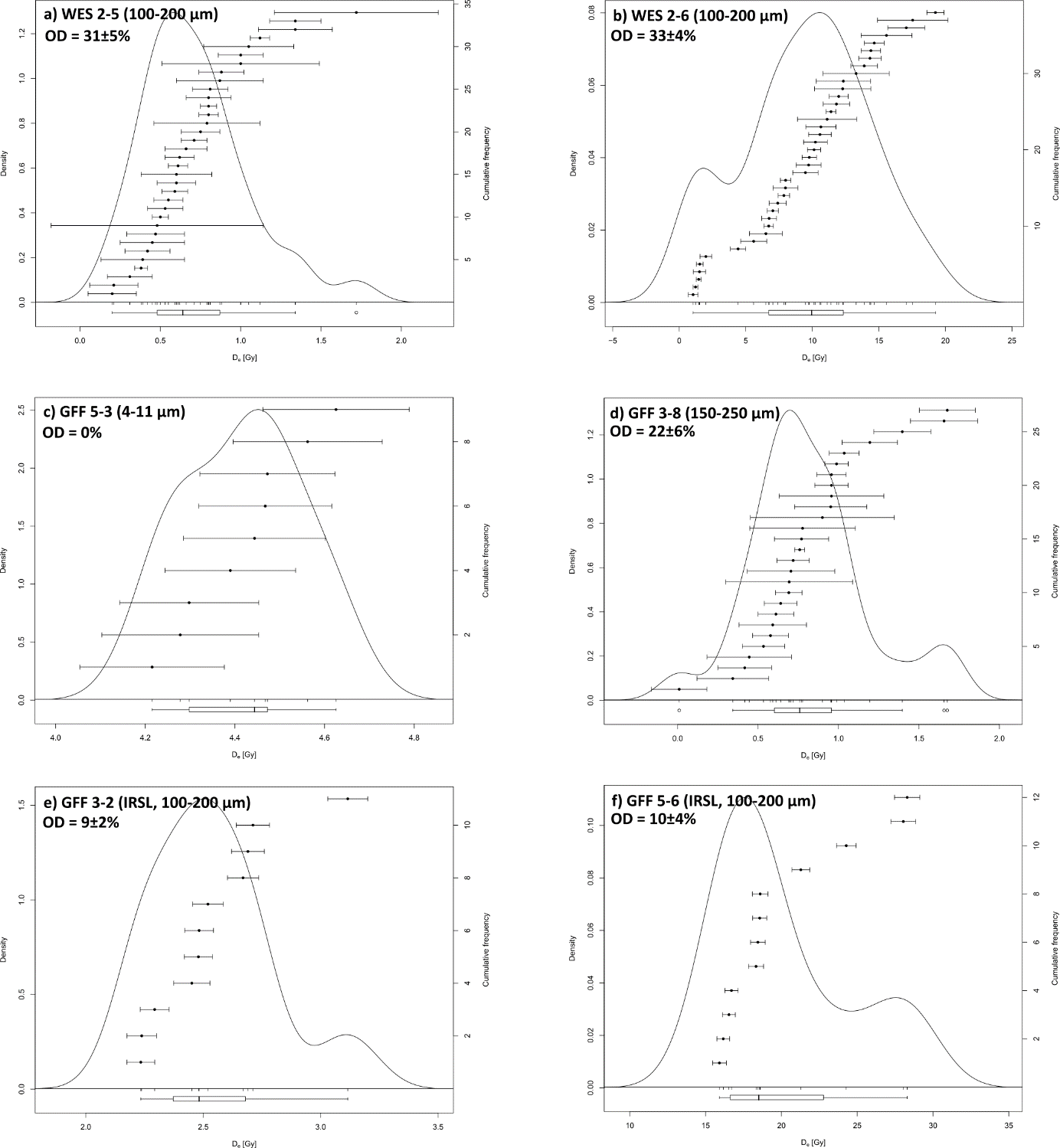


*Fig. S9: Equivalent dose distributions of selected luminescence samples from WES (a, b) and GFF (c-f). For samples from GFF different grain-size fractions (4-11 µm – c, 100-200 µm – d, e, f) and different mineral fractions (quartz - c, d, feldspar IRSL – e, f) are shown.*

Feldspar ages were corrected for fading using the approach of Huntley and Lamothe (2001). For this, we used a mean g-value of 2.2±0.3% decade^-1^ for all samples (sample-specific values vary between 1.9 and 2.5 % decade^-1^), which we determined following the approach of Auclair et al. (2003). Data related to burial dose determination are summarized in Table S5 for all samples.

*Tab. S5: Summary of dose and age data for all samples used in this study.*

| **Sample** | **Lab-ID** | **Depth  (cm)** | **Min.*** | **Grain size (µm)** | **N**† | **OD**§ **(%)** | **Dose  (Gy)** | **Dose rate (Gy ka^-1^)** | **Age  (ka)** | **Age cor.** ^#^ **(ka)** |
| --- | --- | --- | --- | --- | --- | --- | --- | --- | --- | --- |
| WES 2-1 | C-L5658 | 7 | Q | 100-200 | 37/88 | 29±21 | 0.06±0.01 | 1.22±0.05 | 0.05±0.01 | - |
| WES 2-3 | C-L5660 | 34 | Q | 100-200 | 37/95 | 27±7 | 0.30±0.02 | 1.24±0.05 | 0.24±0.02 | - |
| WES 2-4 | C-L5661 | 45 | Q | 100-200 | 29/45 | 31±6 | 0.61±0.04 | 1.18±0.05 | 0.52±0.04 | - |
| WES 2-5 | C-L5662 | 58 | Q | 100-200 | 35/105 | 31±5 | 0.71±0.05 | 1.14±0.05 | 0.62±0.05 | - |
| WES 2-6 | C-L5663 | 70 | Q | 100-200 | 38123 | 33±4 | 10.4±0.6 | 1.14±0.05 | 9.1±0.7 | - |
|  |  |  | Q | 100-200 |  |  | 1.5±0.2** |  | 1.3±0.2** | - |
| WES 2-7 | C-L5664 | 82 | Q | 100-200 | 36/119 | 12±2 | 13.8±0.4 | 1.10±0.05 | 12.5±0.6 | - |
| GFF 3-1 | C-L5130 | 27 | Q | 4-11 | 7/8 | 0 | 2.47±0.04 | 4.17±0.16 | 0.70±0.04 | - |
| GFF 3-2 | C-L5131 | 45 | Q | 4-11 | 6/10 | 0 | 2.94±0.10 | 3.92±0.16 | 0.81±0.05 | - |
|  |  |  |  | 150-250 | 26/48 | 27±4 | 2.16±0.12 | 2.98±0.12 | 0.73±0.05 | - |
|  |  |  | KF | 100-200 | 11/12 | 9±2 | 2.52±0.07 | 3.71±0.23 | 0.68±0.05 | 0.80±0.06 |
| GFF 3-3 | C-L5132 | 65 | Q | 4-11 | 9/10 | 5±1 | 2.59±0.05 | 4.01±0.16 | 0.70±0.06 | - |
|  |  |  |  | 150-250 | 19/48 | 14±3 | 2.01±0.08 | 3.07±0.12 | 0.67±0.04 | - |
| GFF 3-4 | C-L5133 | 76 | Q | 4-11 | 9/12 | 0 | 2.63±0.08 | 3.69±0.14 | 0.72±0.06 | - |
|  |  |  |  | 150-250 | 27/72 | 16±3 | 1.96±0.08 | 3.06±0.13 | 0.64±0.04 | - |
| GFF 3-5 | C-L5134 | 87 | Q | 4-11 | 13/14 | 0 | 2.28±0.03 | 3.79±0.15 | 0.59±0.04 | - |
|  |  |  |  | 150-250 | 19/48 | 14±3 | 1.82±0.07 | 3.22±0.13 | 0.57±0.03 | - |
| GFF 3-6 | C-L5135 | 103 | Q | 4-11 | 6/6 | 0 | 1.94±0.04 | 3.93±0.15 | 0.51±0.03 | - |
|  |  |  |  | 150-250 | 21/72 | 12±3 | 1.74±0.05 | 3.12±0.13 | 0.56±0.03 | - |
| GFF 3-7 | C-L5136 | 117 | Q | 4-11 | 6/8 | 5±4 | 1.50±0.05 | 3.7±0.14 | 0.41±0.02 | - |
|  |  |  |  | 150-250 | 21/72 | 20±2 | 0.95±0.05 | 3.04±0.12 | 0.31±0.02 | - |
| GFF 3-8 | C-L5137 | 150 | Q | 4-11 | 16/16 | 6±3 | 1.22±0.03 | 3.73±0.14 | 0.30±0.01 | - |
|  |  |  |  | 150-250 | 14/48 | 22±6 | 0.92±0.07 | 3.28±0.14 | 0.28±0.02 | - |
| GFF 3-9 | C-L5138 | 193 | Q | 4-11 | 6/8 | 8±4 | 0.95±0.04 | 3.62±0.13 | 0.24±0.02 | - |
| GFF 3-10 | C-L5139 | 222 | Q | 4-11 | 6/6 | 0 | 0.79±0.03 | 3.55±0.13 | 0.19±0.01 | - |
|  |  |  |  | 150-250 | 15/48 | 37±9 | 0.47±0.05 | 3.42±0.14 | 0.14±0.02 | - |
|  |  |  | KF | 100-200 | 11/12 | 23±5 | 0.67±0.05 | 4.19±0.25 | 0.16±0.02 | 0.19±0.02 |
| GFF 5-1 | C-L5757 | 178 | Q | 4-11 | 9/9 | 0 | 4.4±0.1 | 3.76±0.15 | 1.2±0.1 | - |
| GFF 5-2 | C-L5758 | 195 | Q | 4-11 | 9/9 | 0 | 7.7±0.1 | 3.76±0.15 | 2.1±0.1 | - |
| GFF 5-3 | C-L5991 | 220 | Q | 4-11 | 17/17 | 0 | 8.8±0.3 | 3.76±0.15 | 2.3±0.1 | - |
| GFF 5-4 | C-L5759 | 238 | Q | 4-11 | 9/9 | 0 | 10.7±0.3 | 3.76±0.15 | 2.9±0.1 | - |
| GFF 5-5 | C-L5760 | 247 | Q | 4-11 | 12/12 | 0 | 13.3±0.3 | 3.76±0.15 | 3.6±0.2 | - |
|  |  |  | KF | 100-200 | 12/12 | 20±4 | 19.7±1.1 | 3.73±0.23 | 5.3±0.4 | 6.4±0.6 |
| GFF 5-6 | C-L5708 | 397 | Q | 4-11 | 9/9 | 0 | 48.5±1.1 | 3.95±0.15 | 12.3±0.5 | - |
|  |  |  | KF | 100-200 | 11/12 | 10±4 | 37.7±1.0 | 3.73±0.23 | 10.1±0.8 | 12.7±1.1 |
| GFF 5-7 | C-L5709 | 486 | Q | 4-11 | 10/10 | 6±3 | 59.3±1.7 | 3.49±0.13 | 16.1±0.8 | - |

* Min = mineral with Q – quartz and KF – potassium feldspar

† N – number of accepted versus measured aliquots

§ OD – over-dispersion

# Age cor – age corrected for fading using the mean g-value calculated from all samples

** Dose and age resulting from MAM on the younger population of the sample

While all quartz ages except from sample WES 2-6, which is affected by mixing of two different substrates, are well bleached, feldspar luminescence signals show signs of incomplete IRSL signal resetting for sample GFF 5-5, taken from pre-Roman colluvium. This is demonstrated by an IRSL age approximately twice as high as the associated quartz age, while for all other measured samples at GFF quartz and feldspar ages agree within uncertainties (Fig. S10).


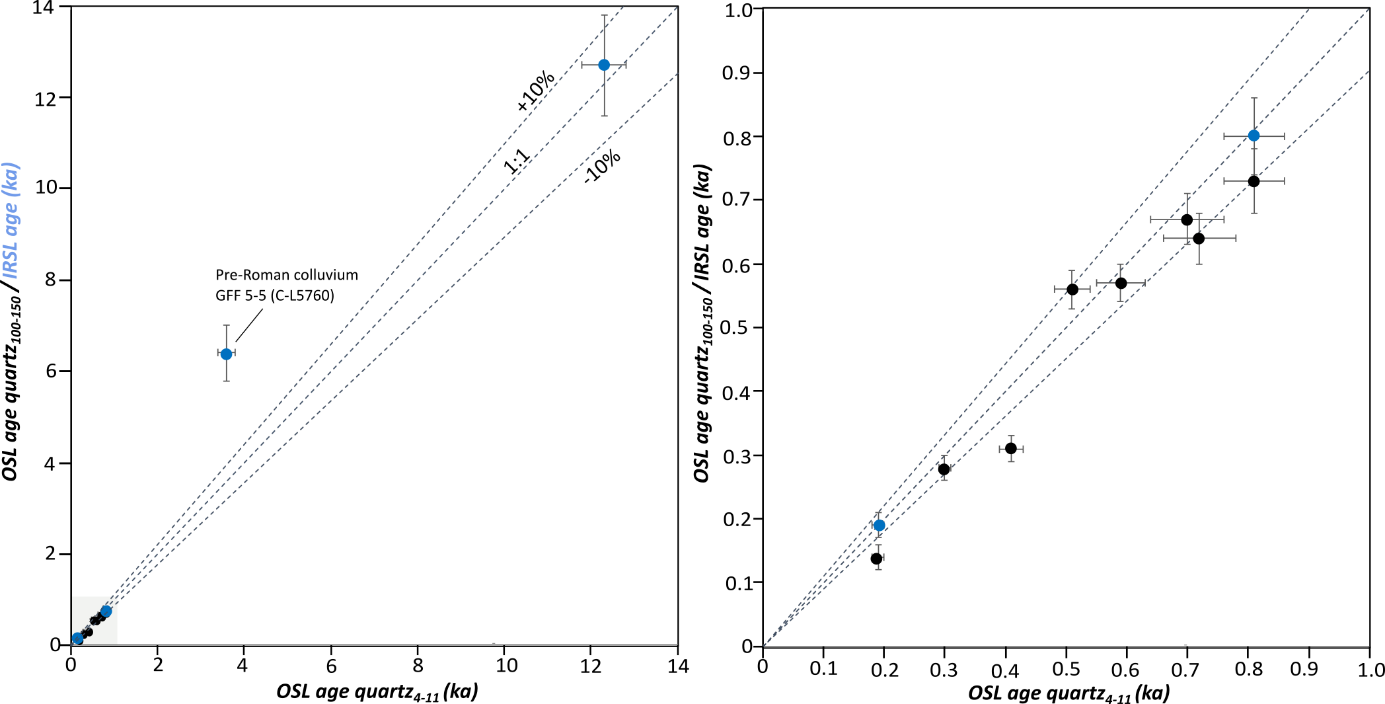


*Fig. S10: Comparison between fine-grain quartz OSL ages and ages derived on the coarse-grain fraction, both feldspar IRSL (blue) and quartz OSL (black) for GFF (left). Zoom to data for the last 1000 years (right).*

***S5. Portable luminescence reader measurements and analysis***

***S5.1. Protocol and measurements***

All portable OSL reader (pOSL) analyses were carried out under dimmed red-light conditions in the CLL. Bulk sediment samples were dried at 50 °C and gently pestled. Normalized volumes of 1 cm³ were filled in petry dishes and measured with a SUERC portable luminescence reader (Sanderson and Murphy, 2010) by subsequently stimulating with infrared (IRSL signal) and blue LEDs (BSL signal) for 60 s each. Both signals show comparable trends for the samples in this study. However, IRSL signals are derived solely from feldspar minerals, while BSL signals originate from both quartz and feldspar and are, thus, more sensitive to variations of mineral composition between samples. Therefore, we used the IRSL signal for all pOSL analyses. The pOSL signals were calculated by summing the first 10 s of stimulation. We performed between two and three measurements for each sample and used the arithmetic mean of all replicates for further analyses (Tab. S6, Tab. S7, Tab. S8). More aliquots (up to 20) were measured for calibration samples GFF 3-8, 5-4, 5-6 and 5-7. These samples were selected to investigate internal pOSL signal scatter for different age ranges of the stratigraphy.

*Tab. S6: Mean pOSL signals and pOSL-based ages for WES 1.*

| **Sample** | **Unit** | **Depth (cm)** | **IRSL_mean_ (cts)** | **IRSL_ER (15%)_ (cts)** | **Equ.** | **Age_pOSL_ (ka)** | **Age_Er (15%)_ (ka)** |
| --- | --- | --- | --- | --- | --- | --- | --- |
| WES 1-1 | **Plaggen soil** | 2.4 | 45 | 7 | **1** | 0.101 | 0.015 |
| WES 1-2 |  | 4.8 | 28 | 4 |  | 0.069 | 0.010 |
| WES 1-3 |  | 7.2 | 38 | 6 |  | 0.088 | 0.013 |
| WES 1-4 |  | 9.6 | 16 | 2 |  | 0.044 | 0.007 |
| WES 1-5 |  | 12 | 28 | 4 |  | 0.069 | 0.010 |
| WES 1-6 |  | 14.4 | 15 | 2 |  | 0.040 | 0.006 |
| WES 1-7 |  | 16.8 | 82 | 12 |  | 0.168 | 0.025 |
| WES 1-8 |  | 19.2 | 77 | 12 |  | 0.159 | 0.024 |
| WES 1-9 |  | 21.6 | 33 | 5 |  | 0.079 | 0.012 |
| WES 1-10 |  | 24 | 72 | 11 |  | 0.150 | 0.022 |
| WES 1-11 |  | 26.4 | 39 | 6 |  | 0.090 | 0.013 |
| WES 1-12 |  | 28.8 | 82 | 12 |  | 0.167 | 0.025 |
| WES 1-13 |  | 31.2 | 64 | 10 |  | 0.136 | 0.020 |
| WES 1-14 |  | 33.6 | 53 | 8 |  | 0.116 | 0.017 |
| WES 1-15 |  | 36 | 114 | 17 |  | 0.219 | 0.033 |
| WES 1-16 |  | 38.4 | 176 | 26 |  | 0.315 | 0.047 |
| WES 1-17 |  | 40.8 | 238 | 36 |  | 0.404 | 0.061 |
| WES 1-18 |  | 43.2 | 209 | 31 |  | 0.362 | 0.054 |
| WES 1-19 |  | 45.6 | 401 | 60 |  | 0.624 | 0.094 |
| WES 1-20 |  | 48 | 307 | 46 |  | 0.499 | 0.075 |
| WES 1-21 |  | 50.4 | 369 | 55 |  | 0.581 | 0.087 |
| WES 1-22 |  | 52.8 | 286 | 43 |  | 0.470 | 0.071 |
| WES 1-23 |  | 55.2 | 299 | 45 |  | 0.489 | 0.073 |
| WES 1-24 |  | 57.6 | 367 | 55 |  | 0.580 | 0.087 |
| WES 1-25 |  | 60 | 395 | 59 |  | 0.616 | 0.092 |
| WES 1-26 |  | 62.4 | 1282 | 192 |  | 1.635 | 0.245 |
| WES 1-27 |  | 64.8 | 943 | 141 |  | 1.267 | 0.190 |
| WES 1-28 |  | 67.2 | 2609 | 391 |  | 2.947 | 0.442 |
| WES 1-29 |  | 69.6 | 4580 | 687 |  | 4.701 | 0.705 |
| WES 1-30 | **Cover sand** | 72 | 11038 | 1656 | **1** | 9.750 | 1.463 |
| WES 1-31 |  | 74.4 | 21766 | 3265 |  | 17.124 | 2.569 |
| WES 1-32 |  | 76.8 | 25554 | 3833 |  | 19.562 | 2.934 |
| WES 1-33 |  | 79.2 | 17927 | 2689 |  | 14.578 | 2.187 |
| WES 1-34 |  | 81.6 | 16947 | 2542 |  | 13.915 | 2.087 |
| WES 1-35 |  | 84 | 16250 | 2437 |  | 13.438 | 2.016 |
| WES 1-36 |  | 86.4 | 17090 | 2564 |  | 14.012 | 2.102 |

*Tab. S7: Mean pOSL signals and pOSL-based ages for GFF 5.*

| **Sample** | **Depth (cm)** | **Unit** | **IRSL_mean_ (cts)** | **IRSL_ER (15%)_ (cts)** | **Equ.** | **Age_pOSL_ (ka)** | **Age_Er (15%)_ (ka)** |
| --- | --- | --- | --- | --- | --- | --- | --- |
| GFF 5-1 | 1 | **Colluvium** | 204 | 31 | **2** | -0.015 | -0.002 |
| GFF 5-2 | 11 |  | 89 | 13 |  | -0.119 | -0.018 |
| GFF 5-3 | 21 |  | 117 | 18 |  | -0.093 | -0.014 |
| GFF 5-4 | 31 |  | 95 | 14 |  | -0.114 | -0.017 |
| GFF 5-5 | 41 |  | 144 | 22 |  | -0.069 | -0.010 |
| GFF 5-6 | 51 |  | 107 | 16 |  | -0.102 | -0.015 |
| GFF 5-7 | 61 |  | 242 | 36 |  | 0.020 | 0.003 |
| GFF 5-8 | 71 |  | 393 | 59 |  | 0.156 | 0.023 |
| GFF 5-9 | 81 |  | 639 | 96 |  | 0.376 | 0.056 |
| GFF 5-10 | 91 |  | 828 | 124 |  | 0.544 | 0.082 |
| GFF 5-11 | 101 |  | 742 | 111 |  | 0.468 | 0.070 |
| GFF 5-12 | 111 |  | 834 | 125 |  | 0.549 | 0.082 |
| GFF 5-13 | 121 |  | 812 | 122 |  | 0.530 | 0.079 |
| GFF 5-14 | 131 |  | 917 | 138 |  | 0.623 | 0.093 |
| GFF 5-15 | 141 |  | 872 | 131 |  | 0.583 | 0.087 |
| GFF 5-16 | 151 |  | 872 | 131 |  | 0.583 | 0.087 |
| GFF 5-17 | 161 |  | 689 | 103 |  | 0.421 | 0.063 |
| GFF 5-18 | 171 |  | 751 | 113 |  | 0.476 | 0.071 |
| GFF 5-19 | 173 |  | 980 | 147 |  | 0.678 | 0.102 |
| GFF 5-20 | 175 |  | 1220 | 183 |  | 0.888 | 0.133 |
| GFF 5-21 | 177 |  | 1173 | 176 |  | 0.847 | 0.127 |
| GFF 5-22 | 179 |  | 1402 | 210 |  | 1.046 | 0.157 |
| GFF 5-23 | 181 |  | 1265 | 190 |  | 0.927 | 0.139 |
| GFF 5-24 | 183 |  | 1714 | 257 |  | 1.313 | 0.197 |
| GFF 5-25 | 185 |  | 2556 | 383 |  | 2.020 | 0.303 |
| GFF 5-26 | 187 |  | 2893 | 434 |  | 2.297 | 0.345 |
| GFF 5-27 | 189 |  | 2649 | 397 |  | 2.098 | 0.315 |
| GFF 5-28 | 191 |  | 2570 | 386 |  | 2.032 | 0.305 |
| GFF 5-29 | 201 |  | 2554 | 383 |  | 2.019 | 0.303 |
| GFF 5-30 | 211 |  | 2619 | 393 |  | 2.072 | 0.311 |
| GFF 5-31 | 213 | **Colluvium (pre Roman)** | 3241 | 486 | **3** | 2.141 | 0.321 |
| GFF 5-32 | 215 |  | 3261 | 489 |  | 2.145 | 0.322 |
| GFF 5-33 | 217 |  | 3746 | 562 |  | 2.234 | 0.335 |
| GFF 5-34 | 219 |  | 3895 | 584 |  | 2.262 | 0.339 |
| GFF 5-35 | 221 |  | 4387 | 658 |  | 2.352 | 0.353 |
| GFF 5-36 | 223 |  | 4615 | 692 |  | 2.394 | 0.359 |
| GFF 5-37 | 225 |  | 4995 | 749 |  | 2.464 | 0.370 |
| GFF 5-38 | 227 |  | 5581 | 837 |  | 2.572 | 0.386 |
| GFF 5-39 | 229 |  | 5288 | 793 |  | 2.518 | 0.378 |
| GFF 5-40 | 231 |  | 4110 | 617 |  | 2.301 | 0.345 |
| GFF 5-41 | 233 |  | 5743 | 861 |  | 2.602 | 0.390 |
| GFF 5-42 | 235 |  | 7202 | 1080 |  | 2.870 | 0.431 |
| GFF 5-43 | 237 |  | 7170 | 1076 |  | 2.865 | 0.430 |
| GFF 5-44 | 239 |  | 7744 | 1162 |  | 2.970 | 0.446 |
| GFF 5-45 | 241 |  | 7094 | 1064 |  | 2.851 | 0.428 |
| GFF 5-46 | 243 |  | 9381 | 1407 |  | 3.271 | 0.491 |
| GFF 5-47 | 245 |  | 10292 | 1544 |  | 3.439 | 0.516 |
| GFF 5-48 | 247 |  | 11155 | 1673 |  | 3.598 | 0.540 |
| GFF 5-49 | 249 |  | 10797 | 1620 |  | 3.532 | 0.530 |
| GFF 5-50 | 251 | **Loess** | 17534 | 2630 | **2** | 11.369 | 1.705 |
| GFF 5-51 | 261 |  | 18648 | 2797 |  | 11.869 | 1.780 |
| GFF 5-52 | 271 |  | 20070 | 3010 |  | 12.475 | 1.871 |
| GFF 5-53 | 281 |  | 26056 | 3908 |  | 14.688 | 2.203 |
| GFF 5-54 | 291 |  | 21360 | 3204 |  | 12.997 | 1.950 |
| GFF 5-55 | 301 |  | 22487 | 3373 |  | 13.432 | 2.015 |
| GFF 5-56 | 311 |  | 24403 | 3660 |  | 14.128 | 2.119 |
| GFF 5-57 | 321 |  | 24699 | 3705 |  | 14.231 | 2.135 |
| GFF 5-58 | 331 |  | 23301 | 3495 |  | 13.734 | 2.060 |
| GFF 5-59 | 341 |  | 21518 | 3228 |  | 13.059 | 1.959 |
| GFF 5-60 | 351 |  | 23342 | 3501 |  | 13.749 | 2.062 |
| GFF 5-61 | 361 |  | 23500 | 3525 |  | 13.806 | 2.071 |
| GFF 5-62 | 371 |  | 22736 | 3410 |  | 13.525 | 2.029 |
| GFF 5-63 | 381 |  | 19066 | 2860 |  | 12.051 | 1.808 |
| GFF 5-64 | 391 |  | 19537 | 2931 |  | 12.252 | 1.838 |
| GFF 5-65 | 401 |  | 21355 | 3203 |  | 12.995 | 1.949 |
| GFF 5-66 | 411 |  | 24481 | 3672 |  | 14.155 | 2.123 |
| GFF 5-67 | 421 |  | 33676 | 5051 |  | 16.846 | 2.527 |
| GFF 5-68 | 431 |  | 25179 | 3777 |  | 14.395 | 2.159 |
| GFF 5-69 | 441 |  | 28752 | 4313 |  | 15.527 | 2.329 |
| GFF 5-70 | 451 |  | 25701 | 3855 |  | 14.571 | 2.186 |
| GFF 5-71 | 461 |  | 23873 | 3581 |  | 13.941 | 2.091 |
| GFF 5-72 | 471 |  | 28997 | 4350 |  | 15.598 | 2.340 |
| GFF 5-73 | 481 |  | 32297 | 4845 |  | 16.502 | 2.475 |
| GFF 5-74 | 491 |  | 29402 | 4410 |  | 15.716 | 2.357 |

*Tab. S8: Mean pOSL signals and pOSL-based ages for GFF 3.*

| **Sample** | **Unit** | **Depth (cm)** | **IRSL_mean_ (cts)** | **IRSL_ER (15%)_ (cts)** | **Equ.** | **Age_pOSL_ (ka)** | **Age_Er (15%)_ (ka)** |
| --- | --- | --- | --- | --- | --- | --- | --- |
| GFF 3-1 | **Colluvium** | **1** | 119 | 18 | 2 | -0.09 | -0.01 |
| GFF 3-2 |  | 4 | 122 | 18 |  | -0.09 | -0.01 |
| GFF 3-3 |  | 7 | 137 | 21 |  | -0.08 | -0.01 |
| GFF 3-4 |  | 9 | 151 | 23 |  | -0.06 | -0.01 |
| GFF 3-5 |  | 12 | 181 | 27 |  | -0.04 | -0.01 |
| GFF 3-6 |  | 15 | 176 | 26 |  | -0.04 | -0.01 |
| GFF 3-7 |  | 17 | 242 | 36 |  | 0.02 | 0.00 |
| GFF 3-8 |  | 20 | 216 | 32 |  | 0.00 | 0.00 |
| GFF 3-9 |  | 23 | 245 | 37 |  | 0.02 | 0.00 |
| GFF 3-10 |  | 25 | 196 | 29 |  | -0.02 | 0.00 |
| GFF 3-11 |  | 28 | 196 | 29 |  | -0.02 | 0.00 |
| GFF 3-12 |  | 30 | 230 | 35 |  | 0.01 | 0.00 |
| GFF 3-13 |  | 33 | 218 | 33 |  | 0.00 | 0.00 |
| GFF 3-14 |  | 36 | 323 | 48 |  | 0.09 | 0.01 |
| GFF 3-15 |  | 38 | 238 | 36 |  | 0.02 | 0.00 |
| GFF 3-16 |  | 41 | 228 | 34 |  | 0.01 | 0.00 |
| GFF 3-17 |  | 44 | 280 | 42 |  | 0.05 | 0.01 |
| GFF 3-18 |  | 46 | 419 | 63 |  | 0.18 | 0.03 |
| GFF 3-19 |  | 49 | 396 | 59 |  | 0.16 | 0.02 |
| GFF 3-20 |  | 52 | 490 | 74 |  | 0.24 | 0.04 |
| GFF 3-21 |  | 54 | 463 | 69 |  | 0.22 | 0.03 |
| GFF 3-22 |  | 57 | 396 | 59 |  | 0.16 | 0.02 |
| GFF 3-23 |  | 60 | 410 | 62 |  | 0.17 | 0.03 |
| GFF 3-24 |  | 62 | 406 | 61 |  | 0.17 | 0.03 |
| GFF 3-25 |  | 65 | 456 | 68 |  | 0.21 | 0.03 |
| GFF 3-26 |  | 68 | 538 | 81 |  | 0.29 | 0.04 |
| GFF 3-27 |  | 70 | 503 | 75 |  | 0.26 | 0.04 |
| GFF 3-28 |  | 73 | 650 | 98 |  | 0.39 | 0.06 |
| GFF 3-29 |  | 75 | 611 | 92 |  | 0.35 | 0.05 |
| GFF 3-30 |  | 78 | 497 | 75 |  | 0.25 | 0.04 |
| GFF 3-31 |  | 81 | 751 | 113 |  | 0.48 | 0.07 |
| GFF 3-32 |  | 83 | 812 | 122 |  | 0.53 | 0.08 |
| GFF 3-33 |  | 86 | 734 | 110 |  | 0.46 | 0.07 |
| GFF 3-34 |  | 89 | 699 | 105 |  | 0.43 | 0.06 |

***S5.2. pOSL-age transfer functions***

We used Sigmaplot version 15.0 to perform regression analyses and fitting of pOSL calibration data to derive transfer functions. For WES, data were best fit with a power function of the form Age_pOSL_ (years) = 4.4 x pOSL^0.82^ (function 1). The regression has an R² of 0.99 and the p-values of both parameters are below 0.05 (a=0.0015, b<0.0001). For GFF, the data was fit after excluding samples GFF 5-3, GFF 5-4 and GFF 5-5 as outliers, since they suffer from incompletely reset feldspar IRSL signals that lead to over-estimated pOSL signals. The reduced calibration dataset was best fit with a saturating exponential function of the form Age_pOSL_ (years) = -200+(23,200 x (1-e^-0.00004 x pOSL^) (function 2). The regression has an R² of 0.99 and the p-values of all three parameters are below 0.05 (y_0_=0.0004, a<0.0001, b<0.0001). The poorly bleached samples GFF 5-3, 5-4 and 5-5 were fit by a linear function of the form Age_pOSL_ (years) = 0.2 x pOSL + 1,540 (function 3), with an R^2^ of 0.99 and p values of <0.05 as well.

***S5.3. Error estimation of pOSL-based ages***

Errors for the pOSL signals include uncertainties for (i) the sample-internal pOSL signal scatter of 4.5% (GFF) and 14.8% (WES) determined on representative samples with 17-19 pOSL replicates each (Tabs S9, S10); as well as (ii) inter-sample scatter of dose rates of 5.3% (GFF) and 5.9% (WES) (Tab. S4); and (iii) inter-sample scatter of luminescence sensitivity of 13.6% (GFF) and 12% (WES) determined on the calibration samples. Altogether, this adds up to a relative error of 15-20% for samples from both sites. As a mutual uncertainty for samples from both sites we used a value of 15%. Finally, an instrumental counting error of 25 counts was added to all samples. Relative 1-sigma errors based on the pOSL measurements were added in quadrature and multiplied with the age estimate of the sample to derive the sample-specific error in years (Eq. S1), which amounted to 15-32% of the age estimate.

$age_{pOSL, error}=\sqrt{\left( {0.15}^{2}+\left( \frac{25}{mean_{IRSL}} \right)^{2} \right)}*age_{pOSL}$ (Eq. S1)

*Tab. S9: Internal pOSL signal scatter for GFF samples. Values were determined by measuring 17-19 pOSL aliquots of the same sample and calculating their reative standard deviations (RSD). plow*

|  | **GFF 5-4** | | **GFF 5-7** | |
| --- | --- | --- | --- | --- |
| **Aliquot** | **IRSL (cts)** | **IRSL_Er_ (cts)** | **IRSL (cts)** | **IRSL_Er_ (cts)** |
| 1 | 61549 | 246 | 79792 | 284 |
| 2 | 66089 | 257 | 79863 | 280 |
| 3 | 55034 | 234 | 76747 | 277 |
| 4 | 63677 | 251 | 85178 | 293 |
| 5 | 60536 | 246 | 80236 | 283 |
| 6 | 60434 | 246 | 80142 | 283 |
| 7 | 62904 | 251 | 80755 | 285 |
| 8 | 59754 | 245 | 80837 | 283 |
| 9 | 63079 | 251 | 74551 | 271 |
| 10 | 63526 | 250 | 79193 | 282 |
| 11 | 61725 | 249 | 78680 | 278 |
| 12 | 60602 | 246 | 81570 | 282 |
| 13 | 59537 | 245 | 82869 | 289 |
| 14 | 66440 | 259 | 84162 | 290 |
| 15 | 56605 | 237 | 83057 | 289 |
| 16 | 63746 | 253 | 86233 | 294 |
| 17 | 63388 | 252 | 84859 | 292 |
| 18 | - | - | 84104 | 291 |
| 19 | - | - | 73618 | 271 |
| Mean (cts) | **61684** | - | **80866** | - |
| RSD (%) | **4.8%** | - | **4.3%** | - |

*Tab. S10: Internal pOSL signal scatter for WES samples. The mean relative standard deviation (RSD) was calculated based on RSDs of individual samples (based on 3 aliquots per sample) using all samples below the plowplow layer.*

| **Sample** | **RSD (%)** |
| --- | --- |
| WES 1-15 | 1 |
| WES 1-16 | 3 |
| WES 1-17 | 27 |
| WES 1-18 | 25 |
| WES 1-19 | 12 |
| WES 1-20 | 20 |
| WES 1-21 | 24 |
| WES 1-22 | 25 |
| WES 1-23 | 10 |
| WES 1-24 | 33 |
| WES 1-25 | 8 |
| WES 1-26 | 8 |
| WES 1-27 | 10 |
| WES 1-28 | 4 |
| WES 1-29 | 2 |
| WES 1-30 | 4 |
| WES 1-31 | 11 |
| WES 1-32 | 9 |
| WES 1-33 | 3 |
| **Mean RSD (%)** | **14.8%** |

***S5.4. Bayesian age modelling***

We used Bayesian age-depth modelling to constrain the errors of the pOSL age estimates and to resolve any age inversions in the chronologies (Tab. S11). The modelling was performed in Oxcal version 4.4 (Bronk Ramsey, 2009). For both locations, we developed a separate age-depth model. We used Sequence deposition models (Bronk Ramsey, 2008) for the different stratigraphic units identified in the profiles (Figs. S3, S4). Plow layers were implemented as Phase models, as we expected similar ages throughout these well-mixed layers. In between stratigraphic units, we placed double boundaries to allow for non-continuous age-depth trends. In the colluvium and loess of GFF, we placed additional single boundaries at discontinuities in the chronology to allow for changes in deposition rates. pOSL age estimates were implemented as the year before the moment of sampling in 2022, with a corresponding error estimated with Eq. S1 and the sampling depth *z*.

*Tab. S11: Code for the age-depth models in Oxcal for both profiles.*

| **Code for Oxcal model for WES** | **Code for Oxcal model for WGFF, part 1** | **Code for Oxcal model for WGFF, part 2** |
| --- | --- | --- |
| Plot()  {  Sequence("WES")  {  Boundary("Bottom of C");  Sequence("C")  {  C_Date("WES_36", -11585, 2041) { z = 85.2; };  C_Date("WES_35", -11030, 1958) { z = 82.8; };  C_Date("WES_34", -11491, 2027) { z = 80.4; };  C_Date("WES_33", -12131, 2123) { z = 78; };  C_Date("WES_32", -16937, 2844) { z = 75.6; };  C_Date("WES_31", -14587, 2492) { z = 73.2; };  C_Date("WES_30", -7466, 1424) { z = 70.8; };  };  Boundary("Start of C");  Boundary("End of Aap2");  Sequence("Aap2")  {  C_Date("WES_29", -2571, 690) { z = 68.4; };  C_Date("WES_28", -865, 434) { z = 66; };  C_Date("WES_27", 775, 190) { z = 63.6; };  C_Date("WES_26", 415, 243) { z = 61.2; };  C_Date("WES_25", 1414, 99) { z = 58.8; };  C_Date("WES_24", 1449, 94) { z = 56.4; };  C_Date("WES_23", 1539, 83) { z = 54; };  C_Date("WES_22", 1557, 81) { z = 51.6; };  C_Date("WES_21", 1448, 95) { z = 49.2; };  C_Date("WES_20", 1529, 84) { z = 46.8; };  C_Date("WES_19", 1406, 100) { z = 44.4; };  C_Date("WES_18", 1663, 69) { z = 42; };  C_Date("WES_17", 1623, 73) { z = 39.6; };  C_Date("WES_16", 1710, 65) { z = 37.2; };  C_Date("WES_15", 1805, 58) { z = 34.8; };  C_Date("WES_14", 1907, 58) { z = 32.4; };  C_Date("WES_13", 1887, 57) { z = 30; };  C_Date("WES_12", 1856, 57) { z = 27.6; };  };  Boundary("Start of Aap2");  Boundary("End of Aap1");  Phase("Aap1")  {  C_Date("WES_11", 1933, 59) { z = 25.2; };  C_Date("WES_10", 1873, 57) { z = 22.8; };  C_Date("WES_9", 1944, 61) { z = 20.4; };  C_Date("WES_8", 1865, 57) { z = 18; };  C_Date("WES_7", 1856, 57) { z = 15.6; };  C_Date("WES_6", 1983, 69) { z = 13.2; };  C_Date("WES_5", 1954, 62) { z = 10.8; };  C_Date("WES_4", 1979, 68) { z = 8.4; };  C_Date("WES_3", 1935, 60) { z = 6; };  C_Date("WES_2", 1954, 62) { z = 3.6; };  C_Date("WES_1", 1922, 58) { z = 1.2; };  };  Boundary("Start of Aap1");  };  }; | Plot()  {  Sequence("GFF")  {  Boundary("Bottom loess");  Sequence("loess")  {  C_Date("GFF_74", -13693, 2357) { z = 491; };  C_Date("GFF_73", -14479, 2475) { z = 481; };  C_Date("GFF_72", -13575, 2340) { z = 471; };  C_Date("GFF_71", -11918, 2091) { z = 461; };  C_Date("GFF_70", -12548, 2186) { z = 451; };  C_Date("GFF_69", -13504, 2329) { z = 441; };  C_Date("GFF_68", -12372, 2159) { z = 431; };  C_Date("GFF_67", -14823, 2527) { z = 421; };  C_Date("GFF_66", -12132, 2123) { z = 411; };  Boundary(“Loess1”);  C_Date("GFF_65", -10972, 1949) { z = 401; };  C_Date("GFF_64", -10229, 1838) { z = 391; };  C_Date("GFF_63", -10028, 1808) { z = 381; };  C_Date("GFF_62", -11502, 2029) { z = 371; };  C_Date("GFF_61", -11783, 2071) { z = 361; };  C_Date("GFF_60", -11726, 2062) { z = 351; };  C_Date("GFF_59", -11036, 1959) { z = 341; };  C_Date("GFF_58", -11711, 2060) { z = 331; };  C_Date("GFF_57", -12208, 2135) { z = 321; };  C_Date("GFF_56", -12105, 2119) { z = 311; };  C_Date("GFF_55", -11409, 2015) { z = 301; };  C_Date("GFF_54", -10974, 1950) { z = 291; };  C_Date("GFF_53", -12665, 2203) { z = 281; };  C_Date("GFF_52", -10452, 1871) { z = 271; };  C_Date("GFF_51", -9846, 1780) { z = 261; };  C_Date("GFF_50", -9346, 1705) { z = 251; };  };  Boundary("Top loess");  Boundary("Bottom colluvium");  Sequence("Colluvium")  {  Boundary("Colluvium 1");  C_Date("GFF_49", -1509, 530) { z = 249; };  C_Date("GFF_48", -1575, 540) { z = 247; };  C_Date("GFF_47", -1416, 516) { z = 245; };  C_Date("GFF_46", -1248, 491) { z = 243; };  C_Date("GFF_45", -828, 428) { z = 241; };  C_Date("GFF_44", -947, 446) { z = 239; };  C_Date("GFF_43", -842, 430) { z = 237; };  C_Date("GFF_42", -847, 431) { z = 235; };  C_Date("GFF_41", -579, 390) { z = 233; };  C_Date("GFF_40", -278, 345) { z = 231; };  C_Date("GFF_39", -495, 378) { z = 229; };  C_Date("GFF_38", -549, 386) { z = 227; };  C_Date("GFF_37", -441, 370) { z = 225; };  C_Date("GFF_36", -371, 359) { z = 223; };  C_Date("GFF_35", -329, 353) { z = 221; };  C_Date("GFF_34", -239, 340) { z = 219; };  C_Date("GFF_33", -211, 335) { z = 217; };  C_Date("GFF_32", -122, 322) { z = 215; };  C_Date("GFF_31", -118, 322) { z = 213; }; | Boundary("Colluvium 2");  C_Date("GFF_30", -49, 311) { z = 211; };  C_Date("GFF_29", 4, 304) { z = 201; };  C_Date("GFF_28", -9, 306) { z = 191; };  C_Date("GFF_27", -75, 315) { z = 189; };  C_Date("GFF_26", -274, 345) { z = 187; };  C_Date("GFF_25", 3, 304) { z = 185; };  Boundary("Colluvium 3");  C_Date("GFF_24", 710, 198) { z = 183; };  C_Date("GFF_23", 1096, 140) { z = 181; };  C_Date("GFF_22", 977, 158) { z = 179; };  C_Date("GFF_21", 1176, 128) { z = 177; };  C_Date("GFF_20", 1135, 134) { z = 175; };  C_Date("GFF_19", 1345, 103) { z = 173; };  Boundary("Colluvium 4");  C_Date("GFF_18", 1547, 73) { z = 171; };  C_Date("GFF_17", 1602, 65) { z = 161; };  C_Date("GFF_16", 1440, 89) { z = 151; };  C_Date("GFF_15", 1440, 89) { z = 141; };  C_Date("GFF_14", 1400, 95) { z = 131; };  C_Date("GFF_13", 1493, 81) { z = 121; };  C_Date("GFF_12", 1474, 84) { z = 111; };  C_Date("GFF_11", 1555, 72) { z = 101; };  C_Date("GFF_10", 1479, 83) { z = 91; };  Boundary("Colluvium 5");  C_Date("GFF_9", 1647, 58) { z = 81; };  C_Date("GFF_8", 1867, 25) { z = 71; };  C_Date("GFF_7", 2003, 4) { z = 61; };  C_Date("GFF_6", 2125, 28) { z = 51; };  C_Date("GFF_5", 2092, 16) { z = 41; };  C_Date("GFF_4", 2137, 35) { z = 31; };  };  Boundary("Top colluvium");  Boundary("Bottom Ap");  Phase("Ap")  {  C_Date("GFF_3", 2116, 24) { z = 21; };  C_Date("GFF_2", 2142, 38) { z = 11; };  C_Date("GFF_1", 2038, 3) { z = 1; };  };  Boundary("Top Ap");  };  }; |

***S5.5. Calculation of deposition rates***

The ages resulting from the OxCal modelling were used to calculate deposition rates for the anthropogenic parts of the sediment profiles: the plaggen cover of WES (< 60cm) and the colluvium of GFF (< 250 cm). pOSL signals that were marked by OxCal for having a poor fit after modelling (agreement < 60%) were excluded from the rate calculations, as well as the pOSL signals from the plow layer to avoid a bias in rates near the surface due to post-depositional bleaching (Van der Meij et al., 2019).

The OxCal modelling ensured a continuously increasing age with depth, providing positive deposition rates. These deposition rates *r* [cm a^-1^] were calculated by dividing depth increments *∆d* by age increments *∆a* in between two consecutive samples. The corresponding error *σ_r_* was calculated using errors from depth and age increments (Eq. S2).

$r=\frac{\Delta d}{\Delta a}, \sigma_{r}=r*\sqrt{\left( \frac{\sigma_{\Delta d}}{\Delta d} \right)^{2}+\left( \frac{\sigma_{\Delta a}}{\Delta a} \right)^{2}}$ Eq. S2

The error of the depth increments *σ_∆d_* was set to 0, and the error of the age increments *σ_∆a_* was calculated with Eq. S3, using the age errors estimated with Eq. S1 for two consecutive samples *a_i_* and *a_i+1_*.

$\sigma_{\Delta a}=\sqrt{\left( \sigma_{a_{i}} \right)^{2}+\left( \sigma_{a_{i+1}} \right)^{2}}$ Eq. S3

The errors of the deposition rates *σ_r_* are relatively large as a result of the high sampling density and similarity of consecutive ages in the profile. With a lower sampling density, consecutive samples will be less similar and *σ_r_* will be lower, but this will also provide a lower temporal resolution in the chronology and the calculated deposition rates.

Fig. 4 in the manuscript also provides average deposition rates for each sedimentary unit in WES and each historical period in GFF as defined by Gerz (2017). These rates $\bar{r}$were calculated by taking the mean of each rate *r_j_* falling in a unit or period, weighted by the squared rate error *σ_r_*. The corresponding error $\sigma_{\bar{r}}$ was defined as the standard error of the weighted mean (Eq. S4).

$\bar{r}=\frac{\sum_{j=1}^{J} \frac{r_{j}}{\sigma_{r_{j}}^{2}}}{\sum_{j=1}^{J} \frac{1}{\sigma_{r_{j}}^{2}}}, \sigma_{\bar{r}}=\sqrt{\frac{1}{\sqrt{\sum_{j=1}^{J} \frac{1}{\sigma_{r_{j}}^{2}}}}}$ Eq. S4

***References***

Auclair, M., Lamothe, M., and Huot, S., 2003, Measurement of anomalous fading for feldspar IRSL using SAR: Radiation Measurements, v. 37, p. 487–492, doi:<http://dx.doi.org/10.1016/S1350-4487(03)00018-0>.

Blott, S.J., and Pye, K., 2001, GRADISTAT: a grain size distribution and statistics package for the analysis of unconsolidated sediments: Earth Surface Processes and Landforms, v. 26, p. 1237–1248, doi:[10.1002/esp.261](https://doi.org/10.1002/esp.261).

Brinkmann, J., 2002, Räumliche Variabilität von Böden und Bodeneigenschaften auf dem Landwirtschaftlichen Versuchsgut Frankenforst im Pleiser Hügelland: Universität Bonn, 175 p.

Bronk Ramsey, C., 2009, Bayesian Analysis of Radiocarbon Dates: Radiocarbon, v. 51, p. 337–360, doi:[10.1017/S0033822200033865](https://doi.org/10.1017/S0033822200033865).

Bronk Ramsey, C.B., 2008, Deposition models for chronological records: Quaternary Science Reviews, v. 27, p. 42–60, doi:10.1016/j.quascirev.2007.01.019.

Bundesanstalt für Geowissenschaften und Rohstoffe (BGR), 2024, Bodenübersichtskarte v60 1:250.000 (BÜK250), <https://www.bgr.bund.de/DE/Themen/Boden/Informationsgrundlagen/Bodenkundliche_Karten_Datenbanken/BUEK250/buek250_node.html>.

Durcan, J.A., King, G.E., and Duller, G.A.T., 2015, DRAC: Dose Rate and Age Calculator for trapped charge dating: Quaternary Geochronology, v. 28, p. 54–61, doi:<http://dx.doi.org/10.1016/j.quageo.2015.03.012>.

Galbraith, R.F., Roberts, R.G., Laslett, G.M., Yoshida, H., and Olley, J.M., 1999, Optical dating of single and multiple grains of quartz from jinmium rock shelter, northern australia: part i, experimental design and statistical models: Archaeometry, v. 41, p. 339–364.

Geobasis NRW, 2024, Digitales Geländemodell NRW Gitterweite 1m, https://www.opengeodata.nrw.de/produkte/geobasis/hm/dgm1_tiff/dgm1_tiff/

Geologischer Dienst NRW, 2001, Geologische Karte von Nordrhein-Westfalen 1:25000. Blatt 3809 Metelen.

Geologisches Landesamt, 1978, Geologische Karte von Nordrhein-Westfalen 1:25000. Blatt 5209 Siegen.

Gerz, J., 2017, Prähistorische Mensch-Umwelt-Interaktionen im Spiegel von Kolluvien und Befundböden in zwei Löss-Altsiedellandschaften mit unterschiedlicher Boden- und Kulturgeschichte (Schwarzerderegion bei Halle/Saale und Parabraunerderegion Niederrheinische Bucht): Universität zu Köln, 190 p.

Preston, N.J., 2001, Geomorphic Response to Environmental Change: The Imprint of Deforestation and Agricultural Land Use on the Contemporary Landscape of the Pleiser Hügelland, Bonn, Germany: Universität Bonn, 136 p.

Folk, R.L., Ward, W.C., 1957, Brazos River bar: a study in the significance of grain size parameters: Journal of Sedimentary Petrology, v. 27, p. 3–26.

Guérin, G., Mercier, N., and Adamiec, G., 2011, Dose-rate conversion factors: update: Ancient TL, v. 19, p. 5–8.

Huntley, D.J., and Lamothe, M., 2001, Ubiquity of anomalous fading in K-feldspars and the measurement and correction for it in optical dating: Canadian Journal of Earth Sciences, v. 38, p. 1093–1106.

Kreutzer, S., Schmidt, C., DeWitt, R., and Fuchs, M., 2014, The a-value of polymineral fine grain samples measured with the post-IR IRSL protocol: Radiation Measurements, v. 69, p. 18–29, doi:[10.1016/j.radmeas.2014.04.027](https://doi.org/10.1016/j.radmeas.2014.04.027).

Lai, Z.P., Zöller, L., Fuchs, M., and Brückner, H., 2008, Alpha efficiency determination for OSL of quartz extracted from Chinese loess: Radiation Measurements, v. 43, p. 767–770, doi:[10.1016/j.radmeas.2008.01.022](https://doi.org/10.1016/j.radmeas.2008.01.022).

Murray, A.S., and Wintle, A.G., 2000, Luminescence dating of quartz using an improved single-aliquot regenerative-dose protocol: Radiation Measurements, v. 32, p. 57–73, doi:[10.1016/S1350-4487(99)00253-X](https://doi.org/10.1016/S1350-4487(99)00253-X).

Prescott, J.R., and Hutton, J.T., 1994, Cosmic ray contributions to dose rates for luminescence and ESR dating: large depths and long-term time variations: Radiat. Meas., v. 23, p. 497–500.

Sanderson, D.C.W., and Murphy, S., 2010, Using simple portable OSL measurements and laboratory characterisation to help understand complex and heterogeneous sediment sequences for luminescence dating: Quaternary Geochronology, v. 5, p. 299–305, doi:http://dx.doi.org/10.1016/j.quageo.2009.02.001.

Van der Meij, W.M., Reimann, T., Vornehm, V.K., Temme, A.J., Wallinga, J., van Beek, R., and Sommer, M., 2019, Reconstructing rates and patterns of colluvial soil redistribution in agrarian (hummocky) landscapes: Earth Surface Processes and Landforms, v. 44, p. 2408–2422.

Vlaams Planbureau voor Omgeving (VPO), 2017, Digitale bodemkaart van het Vlaams Gewest 2.0 (1:20.000). https://dov.vlaanderen.be/geonetwork/srv/api/records/a1547a01-b9fc-40fa-a2eb-009a39c02c7b.

Wageningen Environmental Research (WENR), 2023, BRO Bodemkaart van Nederland 2023_01 1:50.000. https://basisregistratieondergrond.nl/inhoud-bro/registratieobjecten/modellen/bodemkaart-sgm/

Wallinga, J., Murray, A., and Wintle, A., 2000, The single-aliquot regenerative-dose (SAR) protocol applied to coarse-grain feldspar: Radiation Measurements, v. 32, p. 529–533, doi:<http://dx.doi.org/10.1016/S1350-4487(00)00091-3>.
